# Endogenous mammalian histone H3.3 exhibits chromatin-related functions during development

**DOI:** 10.1186/1756-8935-6-7

**Published:** 2013-04-09

**Authors:** Kelly M Bush, Benjamin TK Yuen, Bonnie L Barrilleaux, John W Riggs, Henriette O’Geen, Rebecca F Cotterman, Paul S Knoepfler

**Affiliations:** 1 Department of Cell Biology and Human Anatomy, University of California Davis School of Medicine, 4303 Tupper Hall, Davis, CA, 95616, USA; 2 Genome Center, University of California Davis School of Medicine, 451 Health Sciences Drive, Davis, CA, 95616, USA; 3 Institute of Pediatric Regenerative Medicine, Shriners Hospital For Children Northern California, 2425 Stockton Blvd., Sacramento, CA, 95817, USA

**Keywords:** Histone variant H3.3, H3f3b, H3f3a, CENP-A, H3.3 Knockout, Chromosome segregation, ChIP-Seq, Histone H3

## Abstract

**Background:**

The histone variant H3.3 plays key roles in regulating chromatin states and transcription. However, the role of endogenous H3.3 in mammalian cells and during development has been less thoroughly investigated. To address this gap, we report the production and phenotypic analysis of mice and cells with targeted disruption of the H3.3-encoding gene, *H3f3b*.

**Results:**

*H3f3b* knockout (KO) mice exhibit a semilethal phenotype traceable at least in part to defective cell division and chromosome segregation. *H3f3b* KO cells have widespread ectopic CENP-A protein localization suggesting one possible mechanism for defective chromosome segregation. KO cells have abnormal karyotypes and cell cycle profiles as well. The transcriptome and euchromatin-related epigenome were moderately affected by loss of *H3f3b* in mouse embryonic fibroblasts (MEFs) with ontology most notably pointing to changes in chromatin regulatory and histone coding genes. Reduced numbers of *H3f3b* KO mice survive to maturity and almost all survivors from both sexes are infertile.

**Conclusions:**

Taken together, our studies suggest that endogenous mammalian histone H3.3 has important roles in regulating chromatin and chromosome functions that in turn are important for cell division, genome integrity, and development.

## Background

The nucleosome is a dynamic structure whose constituent histones undergo a vast number of posttranslational modifications (PTMs), which influence nucleosomal function. In addition, the histones can be entirely replaced by deposition of variant histones carrying their own unique complements of PTMs. For example, canonical histone H3 proteins, H3.1 and H3.2, can be replaced by the histone variant, H3.3. Deposition of H3.3 is thought to produce a nucleosomal state that is less structured and associated with a more transcriptionally active state [1,2]. However H3.3 is not strictly associated with actively transcribed genes and can be found at repressed and poised genes as well [3,4]. One notion consistent with the rather complex relationship between H3.3 and transcriptional states is that H3.3 may be more of a boundary factor for chromatin regulatory domains rather than directly promoting the formation or maintenance of specific transcriptional outcomes [5,6]. Intriguingly, H3.3 has recently been implicated in cellular reprogramming [7] and is theorized to play a key role in global changes in the transcriptome linked to cellular fate.

Another H3 variant, CENP-A is essential for centromere function where a role for histone H3.3 is also implicated [8]. H3.3 may regulate centromeres at least in part by directing appropriate CENP-A protein deposition [9]. While deposition of canonical H3 is replication dependent (RD) and occurs in S phase, H3.3 is expressed [10] and can be deposited either during S phase or in a replication-independent (RI) manner throughout the cell cycle [11]. Nucleosome displacement by the RNA polymerase complex within actively transcribed genes necessitates a higher rate of H3.3-dependent histone replacement throughout different phases of the cell cycle [2,12]. H3.3 is also deposited at chromatin gaps, where it may have a unique function [13]. In addition to its role as a replacement for H3.1 and H3.2, H3.3 also has an antagonistic relationship with histone H1 as a roughly 50% knockdown of H3.3 using RNAi in fly cells substantially increased H1 genomic binding resulting in increased nucleosomal length [14].

The unique functions of the histone H3.3 protein are conferred by the few amino acids by which it varies from canonical histone H3 proteins, and by the distinct expression of H3.3 throughout the cell cycle. H3.3 protein differs by only four and five unique residues from histones H3.2 and H3.1, respectively. Residues S31, A87, I89, and G90 are completely unique to H3.3. Histone H3.3 phosphorylated at Serine31 (H3.3S31P) is enriched at domains bordering centromeres during metaphase and may function in mitosis [15], while amino acids 87, 89, and 90 of canonical H3 play a role in preventing H3.1 and H3.2 from participating in RI deposition [11]. Rescue experiments determined that histone H3.3 and H3 were unable to fully compensate for each other’s loss [16]. A contributing factor to the incomplete rescues may be distinct chaperones used to deposit H3 and H3.3. Two separate chaperone complexes are known to be responsible for H3.3 deposition, and in mouse mesenchymal stem cells (MSCs), are thought to recruit H3.3-H4 dimers at loading centers located in promyelocytic leukemia (PML) bodies [17]. Histone regulator A (HIRA) is a major and critically important H3.3 chaperone, incorporating H3.3 in a replication-independent manner [18,19] into chromatin where active transcription is taking place [3,20]. Meanwhile, the death associated protein, DAXX, and the α-thalassemia X-linked mental retardation protein, ATRX, deposit H3.3 into telomeric and pericentric heterochromatin regions [21,22].

Only two genes, *H3f3a* and *H3f3b* (*H3.3A* and *H3.3B* in *Drosophila*), encode the H3.3 protein, while more than a dozen histone *H3.1* and *H3.2* genes encode the canonical histone H3.1 and H3.2 proteins. The *H3f3a* and *H3f3b* genes also are uniquely located outside canonical histone gene clusters, and their gene structure is distinct from that of canonical histone genes in that they have introns and they also produce mRNAs with polyadenylated tails. When compared to each other, *H3f3a* and *H3f3b* also have distinct gene expression patterns, untranslated regions and promoters [23].

Loss-of-function studies of the two H3.3 genes in *Drosophila*, *H3.3A* and *H3.3B*, have provided key insight into its role in cell biology [16]. While each gene is independently dispensable for fly development, this is likely due to redundancy as double *H3.3* mutants have strong phenotypes including infertility and reduced viability [16,24]. Null flies also exhibit male meiotic defects including impaired chromosome segregation [16]. The biological function of histone H3.3 in higher-order species remains relatively less clear, as most studies have only been able to disrupt one of the two H3.3-encoding genes [25,26] or knockdown expression of H3.3 and its chaperones [27,28]. Knockdown of chromatin-bound H3.3 protein by means of H3.3-directed morpholinos [27], or by the introduction of a dominant-negative form of H3.3 [28] leads to mesodermal developmental defects, possibly due to the inability to obtain sufficient levels of H3.3 to sustain expression of genes involved in differentiation. In mammals, one genetic loss-of-function study has been reported involving a fertility screen-based gene trap of the histone *H3f3a* gene [26]. The gene trap was not a null allele as it reduced *H3f3a* expression only several fold. Nonetheless, mice with the homozygous *H3f3a* gene trap mutation exhibited some evidence of embryonic lethality and infertility even though *H3f3b* is also apparently highly expressed along with *H3f3a* in testes [29]. Another study utilized gene targeting vectors to substitute H3.3 encoding genes with an *H3f3a* or *H3f3b* conditional allele [25], but did not present phenotypic data.

Epitope tagging-based functional genomics studies in MSCs and in mouse embryonic stem (ES) cells have proven of great value in elucidating H3.3 chromatin function [3,4]. These studies indicated that H3.3 is enriched at actively transcribed genes, but not exclusively, and H3.3 enrichment at cell type specific genes changed with differentiation [3,4]. H3.3 was also found enriched in telomeric and particularly pericentromeric heterochromatic regions [21,22,30]. More recently, specific coding mutations in *H3f3a* have been discovered by next generation sequencing in human glioblastoma tumors [31-36]. The mutations consistently changed K27 and G34, but with as yet unknown functional consequences. Intriguingly, the H3.3-related glioblastoma cancers also frequently possess mutations in DAXX or ATRX.

Here, to further investigate the function of H3.3, we report a knockout of *H3f3b* in mice. *H3f3b* KO mice had a semilethal phenotype and survivors were nearly all infertile. The mechanisms underlying these phenotypes converge on cell division, as evidenced by using mouse embryonic fibroblasts (MEFs) as a model. Loss of H3.3 was associated with ectopic CENP-A localization as well as spreading of pericentric heterochromatin. Moderate changes in the transcriptome and epigenome of KO cells were also measured. Overall, our studies provide novel insights into the chromatin and biological functions of histone H3.3.

## Results

### Production and validation of *H3f3b* knockout mice

**Figure 1:**
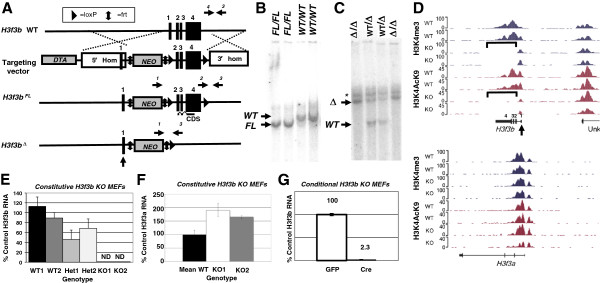
**Generation and validation of conditional, floxed and constitutive knockout (KO) alleles of the*H3f3b*gene.** (**A**) (top) The wildtype (WT) *H3f3b* allele. (second row) The targeting vector contains loxP sites (triangles) that flank exons 2 to 4, a 4.3 kb 5’ arm of homology, a 5.3 kb 3’ arm of homology, diphtheria toxin A (DTA) cassette, and a neomycin (Neo) cassette flanked by frt sites (vertical double arrows). The Neo element allows for positive selection in embryonic stem (ES) cells, while the DTA element permits negative selection in ES cells. (third row) After homologous recombination of the conditional knockout construct, the *H3f3b* gene is expressed until Cre-mediated deletion of exons 2 to 4 (bottom), deleting the entire coding sequence (CDS). (**B**) Southern blotting of *H3f3b* WT and floxed mice using a 3’ probe yields the expected 20 kb and 17 kb bands, respectively validating appropriate gene targeting. (**C**) Southern blotting using a 5’ probe generates the predicted 20 and 10 kb bands for KO and WT alleles in the samples of the indicated genotypes. With this probe a background band (*) was present in all samples. (**D**) Mapped reads from ChIP-Seq assays on KO and WT mouse embryonic fibroblasts (MEFs) 1 and 2 for histone 3 lysine 4 tri-methylation (H3K4me3) and histone 3 lysine 9 acetylation (H3K9ac) indicate precise deletion of the *H3f3b* floxed region with undeleted Exon 1 still exhibiting histone marks. (**E**, **F**) qPCR of *H3f3b* (**E**) and *H3f3a* (**F**) mRNA levels in MEFs of indicated genotypes. (**G**) qPCR assay of *H3f3b* mRNA levels in conditional KO MEFs. Error bars are standard deviations. ND = none detected.

To address H3.3 function in development and chromosome biology, we used homologous recombination to produce a conditional allele of *H3f3b*, *H3f3b*^Fl^, in which the entire coding region contained within exons 2 to 4 is flanked by loxP sites (floxed; Figure 1A). The *H3f3b* locus is 6 to 9 kb from the nearest neighbor genes (Additional file 1: Figure S1A). Genomic PCR genotyping assays for the wildtype (WT), floxed (Fl), and null (*H3f3b*^Δ^) alleles of *H3f3b* were developed (primer locations are indicated in Figure 1A by horizontal arrows) and used to validate the genotypes of the mice (Additional file 1: Figure S1B). *H3f3b*^Fl/WT^ mouse embryonic stem cells were injected into blastocysts to produce chimeras, which were then bred with wildtype mice. Chimeras exhibited germ-line transmission of the *H3f3b*^Fl^ allele producing *H3f3b*^Fl/WT^ pups, and several from one chimera were used as founders. The germline deleter Cre transgene, Zp3-Cre, which is highly expressed early in oocyte development [37], was used to produce *H3f3b*^Δ^ alleles from floxed alleles. Southern blotting of genomic DNA from *H3f3b*^Fl/Fl^, *H3f3b*^WT/WT^ (WT)*,* and *H3f3b*^Δ/Δ^(KO) animals using two separate probes (5’ and 3’) (Additional file 1: Figure S1C) produced the expected bands for each allele (Figure 1B,C). When we conducted chromatin immunoprecipitation-sequencing (ChIP-Seq) for H3K9ac and H3K4me3 in WT and *H3f3b* KO MEFs we found further, independent evidence of precise deletion of the *H3f3b* floxed region (Figure 1D). For both histone H3 lysine 9 acetylation (H3K9ac) and histone H3 lysine 4 tri-methylation (H3K4me3) marks, mapped reads were evident at the *H3f3b* locus corresponding with the transcribed exons in WT cells. Importantly, the floxed region (bracketed) in *H3f3b* KO mice had no reads mapping to the locus, but the H3K9ac and H3K4me3 reads mapping to Exon 1, which should not be deleted in the floxed allele by Cre, remained. As a control, *H3f3a* had the same read profiles in both *H3f3b* WT and KO cells (Figure 1D, bottom). These data argue for a very precise function of our floxed *H3f3b* allele in the presence of Cre.

### Validation of the conditional and constitutive *H3f3b* KO alleles at the mRNA level

Primary MEF cell lines were isolated from E12.5 embryos produced from heterozygous (Het, *H3f3b*^Δ/WT^) intercrosses and stable MEF primary lines of each genotype (WT, Het, and KO) were established. Although we established many lines of *H3f3b* WT and *H3f3b* KO MEFs, in this paper we will focus primarily on those from three specific litters: (1) KO1, KO2 versus WT1, WT2; (2) KO49, KO52 versus WT46, WT48; and (3) KO56 versus WT63. The rationale for choosing and studying MEFs for these initial analyses was that they are a useful cell type that can be reproducibly isolated from midgestational embryos (prior to lethality) for interrogating the KO phenotype. We conducted qPCR on RNA isolated from low passage (<8) MEFs of each line, validating that the *H3f3b*^Δ^ allele was a null as KO MEFs had no detectable *H3f3b* (ND = none detected), and finding, as expected, that Het MEFs exhibited an approximately two-fold reduction in *H3f3b* mRNA levels (Figure 1E). We used qPCR to examine the possibility that there might be compensatory increases in *H3f3a* mRNA levels in *H3f3b* KO MEFs (Figure 1F). Both KO1 and KO2 showed an increase in *H3f3a* mRNA, but KO2 exhibited a more severe reduction in H3.3 protein levels (Additional file 2: Figure S2B, D). We also examined levels of *H3f3a* in uncultured E13.5 embryonic tissue (Additional file 2: Figure S2A). Although we saw a slight relative elevation in *H3f3a* expression in KO 112 and KO 116, it is difficult to say whether or not *H3f3a* has a compensatory effect during development. Overall, data on a number of MEF lines (Figure 1F and Additional file 2: Figure S2B) indicate significant heterogeneity among *H3f3b* KO MEF lines in potential compensatory upregulation of *H3f3a* levels as well as in H3.3 and H3.3S31P protein levels (Additional file 2: Figure S2B-D). For example, one *H3f3b* KO line, KO52, exhibited an approximately three-fold upregulation of *H3f3a* expression. This increase is also apparent at the protein level, with KO52 expressing roughly three fold more H3.3 than littermate KO49 (Additional file 2: Figure S2C, D).

To validate the targeted allele, we produced *H3f3b*^Fl/Fl^ MEFs and transduced them with retrovirus encoding either GFP (control) or a GFP-Cre bicistron. Relative to control, *H3f3b*^Fl/Fl^ MEFs transduced with Cre exhibited a 44-fold reduction in *H3f3b* levels (Figure 1G). Thus, the floxed allele is functional and deleted by Cre.

### The *H3f3b* knockout is semilethal and impairs embryo growth

**Figure 2:**
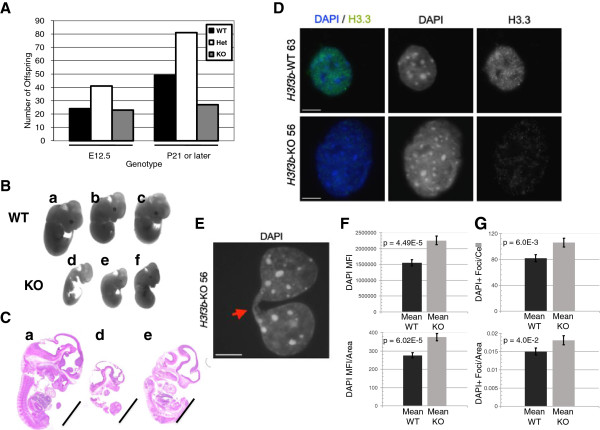
**Loss of*H3f3b*causes a semilethal phenotype and in a subset of embryos strongly impairs overall growth.** (**A**) Ratios of animals of the indicated ages from heterozygous intercrosses indicate substantial lethality among mature knockout (KO) animals.* 0.01 < *P* <0.05 by Chi Square. (**B**, **C**) KO E12.5 embryos exhibit strong reductions in overall growth. Scale bar = 1 mm. (**D**) Wildtype (WT) 63 and KO 56 E12.5 mouse embryonic fibroblasts (MEFs) stained with anti-H3.3 antibodies show higher levels of H3.3 in WT MEFs. (**E**) Chromosomal bridges were observed much more frequently in KO nuclei than in WT nuclei. DAPI staining of a KO 56 nuclei shows representative example of a KO chromosomal bridge. (**F**) *H3f3b* KO MEFs exhibit a statistically significant 1.45 to 1.5-fold increase in DAPI mean fluorescence intensity (MFI) per nuclei, and a 1.21 to 1.36-fold increase in the amount of DAPI MFI per unit of nuclear area. (**G**) *H3f3b* KO MEFs display a significant 1.30 to 1.5-fold increase in the number of pericentric heterochromatic DAPI foci per nuclei, and a significant increase in the number of DAPI-positive foci per unit of nuclear area. Scale bars = 10 um.

Heterozygous animals were present in slightly less than expected Mendelian ratios at maturity suggesting a low level of lethality, but they exhibited no outwardly apparent phenotype and were fertile. When Het mice were intercrossed, surviving mature KO animals arising from those litters were significantly underrepresented (0.01 < *P* <0.05), indicating semilethality (Figure 2A). Such surviving mature KO animals exhibited no outwardly apparent phenotype other than almost uniform infertility in females and complete infertility in all males.

To measure possible embryonic lethality of KO animals, timed matings were conducted using Het parents. Normal Mendelian ratios were observed at E12.5 for KO (Figure 2A), suggesting that the *H3f3b* knockout phenotype predominantly produces lethality in the second half of embryogenesis. However, most KO E12.5 embryos exhibited visibly apparent, abnormal development (Figure 2B,C) indicative of a broad failure of embryo growth. Immunostaining of MEFs using an H3.3-specific antibody [22] revealed both diffuse and punctate H3.3 protein nuclear staining, mostly but not completely outside of heterochromatic foci (Figure 2D). The H3.3 staining was greatly reduced in the *H3f3b* KO MEFs.

### Loss of *H3f3b* leads to chromosomal bridge formation, severe karyotypic abnormalities and endoreduplication

**Figure 3:**
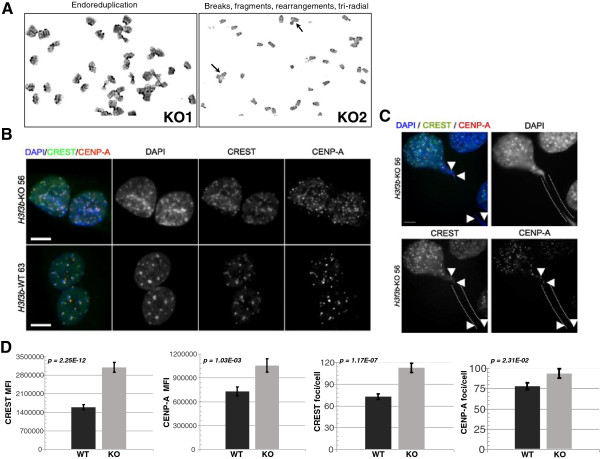
***H3f3b*knockout(KO) mouse embryonic fibroblasts (MEFs) exhibit chromosomal bridges, severe karyotypic abnormalities, and ectopic staining of CENP-A and CREST.** (**A**) Karyotyping revealed a number of types of abnormalities in *H3f3b* KO MEFs not observed in wildtype (WT) MEFs including endoreduplication, chromosomal fragmentation, and tri-radial chromosomes (See also Additional file 3: Figure S3 and Table 1). (**B**) Staining of WT 63 and KO 56 cells with CREST and CENP-A antisera (green and red in merge, respectively). (**C**) KO MEFs with chromosomal bridges had higher levels of CREST and CENPA foci (white arrows). (**D**) Quantification of foci staining for KO 49 and 52 versus WT 46 and 48. An average of 17 confocal sections per nuclei were analyzed using ImageJ software as in this study [43]. Scale bars = 10 um.

*H3f3b* KO MEFs exhibited relatively high levels of mitotic figures and KO mitotic cells had a 17-fold increase in chromosomal bridges (Figure 2E; WT 0.049% versus KO 0.84%, *P* = 0.002), evidence of failure to properly transit mitosis with H3.3 loss-of-function. To examine the potential effects of H3.3 loss-of-function on genomic integrity, two WT and two *H3f3b* KO littermate MEF lines were karyotyped (Figure 3A, Additional file 3: Figure S3A, C, and Table 1). During the analysis, 35 spreads each for WT1 and WT2 and 34 and 33 spreads for KO1 and KO2, were read respectively. The karyotypes of the two WT lines had a mean of 65.7% normal spreads, with only low levels of abnormal chromosome numbers that are sometimes observed in WT MEFs [38] (Additional file 3: Figure S3A). The few WT spreads that had abnormalities predominantly exhibited a low rate of breaks (usually just one or two per spread) (Table 1). The two *H3f3b* KO lines had substantially lower genomic integrity with a mean of 47.8% normal spreads. In addition, the abnormal KO spreads exhibited far higher levels and more severe types of chromosomal aberrations than did the WTs. These changes in the KOs included rearrangements, fragmented ends, multiple tri-radial chromosomes, and intriguingly, in both KO lines, endoreduplication (Figure 3A, Additional file 3: Figure S3A, C, and Table 1). KOs had more than a four-fold increase in spreads with more than two chromosomal abnormalities (Table 1).

**Table 1:** Karyotype analysis for *H3f3b* wildtype (WT) and knockout (KO) mouse embryonic fibroblasts (MEFs)

	**Total spreads**	**Normal spreads**	**Normal ≥ 4N**	**>2 defects**	**Chromosome fragments or breaks**	**Chromatid breaks**	**Triradial**	**Endo-reduplication**	**Rearrangements**
WT1	35	26	5	0	2	0	0	0	0
WT2	35	20	10	2	0	4	0	0	0
Total WT	70	46	15	2	2	4	0	0	0
% Total		65.7%	21.4%	2.9%	2.9%	5.7%	0%	0%	0%
KO1	34	16	6	5	4	4	1	1	1
KO2	33	16	6	3	6	6	2	1	2
Total KO	67	32	12	12	10	10	3	2	3
% Total		47.8%	17.9%	17.9%	14.9%	14.9%	4%	3%	4%

Karyotype analysis done on E12.5 WT (H3f3b^WT/WT^) and KO (H3f3b^Δ/Δ^) MEFs (lines WT1, WT2 and KO1, KO2 respectively). Normal spreads included typical metaphase spread preparation artifacts. Typical artifacts were the loss of 1 to 2 chromosomes per spread. KO MEFs exhibited a greater incidence of abnormalities.

### *H3f3b* removal results in increased pericentric heterochromatin

We utilized DAPI staining to further analyze nuclear structure during interphase in two separate *H3f3b* KO and WT MEFs (*H3f3b* KO lines 49 and 52; *H3f3b* WT lines 46 and 48) (Figure 2F,G). Overall, after examining multiple Z-stack sections through individual nuclei (Figure 2F, top), we found that *H3f3b* KO MEFs prepared identically to *H3f3b* WT MEFs exhibited a 1.5-fold increase (*P* = 4.49E-05, n = 81 *H3f3b* KO and n = 93 *H3f3b* WT nuclei) in DAPI mean fluorescence intensity (MFI). We separately verified the observed increase in DAPI MFI by taking single exposures at lower magnification (n = 356 *H3f3b* WT and n = 309 *H3f3b* KO, *P* = 2.85E-176; data not shown). This increase in DAPI intensity was also significantly higher in *H3f3b* KO MEF nuclei when normalized to nuclear area (*P* = 6.02E-05, Figure 2F, bottom). In addition, we examined the number of intense DAPI foci in *H3f3b* WT and KO MEF nuclei to determine whether the increase in DAPI intensity corresponded to a spread in pericentric heterochromatin [39]. Loss of *H3f3b* resulted in a 1.3-fold increase in the number of pericentric heterochromatin foci (*P* = 6.03E-3, n = 81 *H3f3b* KO and n = 93 *H3f3b* WT) per MEF nuclei (Figure 2G, top). There was also a significant 20% increase in the number of pericentric heterochromatic foci when this data was normalized to nuclear area (*P* = 0.04, Figure 2G, bottom). In agreement with this data, a separate set of *H3f3b* KO nuclei analyzed under identical conditions were found to have a 1.5-fold increase in the number of foci per nuclei (*P* = 6.94E-4, n = 84 *H3f3b* KO and n = 101 *H3f3b* WT nuclei, not shown).

### H3.3 is required for appropriate CENP-A localization

We hypothesized that the changes in nuclear architecture and compromised genomic integrity in *H3f3b* KO MEFs might be due to centromeric dysfunction. To directly examine this possibility, we co-stained interphase cells from two *H3f3b* KO and WT lines with CREST, a human antiserum against the centromere that recognizes a variety of centromeric proteins, and with a specific CENP-A antibody (Figure 3B). *H3f3b* KO MEFs displayed significant increases in kinetochore protein levels by immunocytochemistry. More specifically, KO nuclei generally had increased numbers of both CREST and CENP-A foci corresponding to the observed increase in the number of pericentric heterochromatic foci by DAPI staining (Figure 3B). The majority (but not all) of CREST and CENP-A foci co-localized with DAPI-positive pericentric heterochromatic foci in *H3f3b* KO and WT MEFs. Those KO nuclei exhibiting abnormal mitotic figures and chromosomal bridges had particularly high levels of CREST staining (Figure 3C) including in the bridges themselves. Quantitation of CENP-A and CREST foci as well as MFI by semi-automated ImageJ-based analysis (n = 94 *H3f3b* WT and n = 81 *H3f3b* KO MEF nuclei) indicated that *H3f3b* KO MEFs had significant increases in both CENP-A and CREST foci and total nuclear signal for CENP-A and CREST (Figure 3D). More specifically, *H3f3b* KO MEFs exhibited a 1.95-fold increase in CREST (*P* = 2.25E-12) and a 1.45-fold increase in CENP-A (*P* = 1.03E-03) per nucleus by immunofluorescence. Additionally, *H3f3b* KO MEFs displayed a 1.55-fold increase in the number of CREST-positive foci (*P* = 1.17E-07) and a 1.2-fold increase in the number of CENP-A-positive foci (*P* = 2.31E-02) per nuclei when compared to WT (Figure 3D). *H3f3b* KO MEF nuclei also exhibited higher levels of CREST (*P* = 1.71E-16) and CENP-A (*P* = 8.95E-03) per unit of nuclear area when compared to *H3f3b* WT MEFs (data not shown). To verify these findings, we analyzed a separate set of *H3f3b* WT and KO MEF nuclei under identical conditions (n = 91 *H3f3b* WT and n = 86 *H3f3b* KO), and again found statistically significant increases in the number of CREST- (*P* = 0.017) and CENP-A-positive foci (*P* = 0.004) per nuclei in *H3f3b* KO MEFs (data not shown). Despite these changes, kinetochore staining by CREST still localized to the centromeres of the mouse acrocentric chromosomes during metaphase (Additional file 3: Figure S3D). Moreover, we did not observe gross differences in global histone H3 distribution by Immunocytochemistry-Immunofluorescence (ICC-IF) (Additional file 3: Figure S3E). Because H3.3 was found to incorporate into mouse telomeric chromatin [21,30], we utilized fluorescent *in-situ* hybridization (FISH) to determine whether nuclear integrity could also be affected by dysregulation at the telomere level using a telomere-specific peptide nucleic acid probe. We did not detect a consistent difference in telomere probe hybridization between the *H3f3b* KO and WT MEF nuclei (Additional file 4: Figure S4).

### Loss of *H3f3b* has moderate, but potentially functionally important overall effects on the transcriptome in mouse embryonic fibroblasts

**Figure 4:**
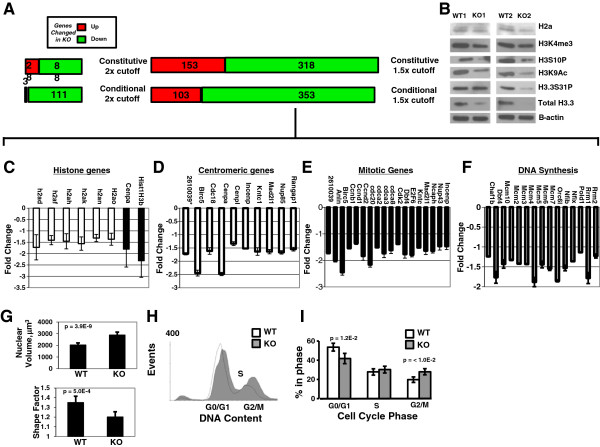
**The ontology of transcriptome changes with loss of*H3f3b*indicates changes in histone, centromere, mitotic, and DNA synthesis genes.** (**A**) RNA isolated from control and *H3f3b* knockout (KO) mouse embryonic fibroblasts (MEFS) (wildtype (WT)1,2 versus KO 1,2) was used for expression microarray studies. Gene expression changes are reported as green and red bars for down and upregulated genes respectively based on the indicated cutoffs for fold changes. (**B**) Immunoblot for H3.3 protein demonstrating strong reductions in total H3.3 and changes in other histone mark protein levels in the two KO MEF lines used for array and ChIP-Seq studies. (**C**) Average gene expression levels of downregulated histones consistent between conditional and constitutive array. (**D**) An ontological cluster of downregulated centromeric genes was evident consistently in *H3f3b* KO MEFs. (**E**) Data from arrays on WT and KO MEFs indicated a very large ontological cluster of mitotic regulatory genes is downregulated in the absence of *H3f3b*. (**F**) DNA synthesis genes downregulated as measured by microarray. (**G**) KO1 MEFs had significantly larger and rounder nuclei compared to littermate MEF WT1. (**H**) Cell cycle analysis by flow cytometry for DNA content on WT MEFs 46 and 48 versus KO MEFs 49 and 52 revealed, when quantitated, as shown in (**I**) a 40% increase in G2/M phase in KO cells. Error bars in (**B-E**, **G**, **I**) are standard deviations. *2610039 is an abbreviation for the 2610039C10Rik gene whose protein product has a mitotic ontology.

An H3.3-specific antibody [22] was used to immunoblot acid extracts from two independent *H3f3b*^⋅^KO MEF lines, KO1 and KO2, which both exhibited greater than 70% reductions in total H3.3 protein relative to WT littermates (Figure 4B and Additional file 2: Figure S2C, D). Two additional KO MEF lines, KO49 and KO52 exhibited similar reductions in H3.3 protein levels as well (Additional file 2: Figure S2C, D). Therefore, we theorized that potential effects of strongly reduced H3.3 levels on the transcriptome should be measurable by array. In light of a recent paper [40] associating microarray results with total RNA levels or production, we analyzed three KO and three WT lines to determine total RNA levels. Although the trend was for KOs to have modestly lower total levels of RNA, there was no statistically significant difference with loss of *H3f3b* (Additional file 5: Figure S5A). Importantly, total H3.3 protein levels did vary between *H3f3b* knockouts, suggesting variable compensatory effects or expression of H3.3 protein by *H3f3a.* One KO line, KO2, had H3.3 levels that were only 5% of WT, while KO1 had 28% of WT levels (Figure 4B and Additional file 2: Figure S2C, D).

To analyze the potential effects of the knockout of *H3f3b* on gene transcription, we isolated RNA from *H3f3b* WT and *H3f3b* KO MEFs derived from littermate E12.5 embryos and conducted expression microarray studies (Figure 4A). We also conducted parallel conditional KO array experiments on *H3f3b*^Fl/Fl^ MEFs transduced with either GFP (control) or GFP-Cre (KO).

Disruption of *H3f3b* and concomitant loss of most H3.3 protein in MEFs caused a moderate detectable change in the global transcriptome (Additional file 6: Table S1; Additional file 7: Table S2 and Figure 4A). Employing a two-fold or greater expression change cutoff in MEFs, only 28 and 3 genes were upregulated by constitutive and conditional loss of *H3f3b*, respectively. Only 88 and 111 genes were consistently downregulated in both of two biological replicates each of constitutive and conditional *H3f3b* KO MEFs, respectively. The most highly downregulated gene in KO MEFs was the *H3f3b* gene itself in every case. Expanding our analysis to genes with expression changes of 1.5 fold or more produced subgroups of 153 and 103 genes upregulated and 318 and 353 genes downregulated with constitutive and conditional deletion of *H3f3b*, respectively.

### The ontology of genes with altered transcription in *H3f3b* knockout mouse embryonic fibroblasts suggests a core relationship of H3.3 with the chromatin and cell cycle regulatory machinery

Although loss of *H3f3b* did not cause pronounced global activation or repression of the overall transcriptome, some of the gene expression changes may be meaningful for the observed phenotypic outcomes. Ontological analysis of genes with altered expression due to loss of *H3f3b* pointed to the following significant functional clusters: histone, centromere-related, mitotic regulatory factor, and DNA synthesis genes (Additional file 8: Table S3, Additional file 9: Table S4 and Figure 4C,E,F, mean of conditional and constitutive KO data; Figure 4D, mean of conditional KO). The ontological cluster of cell cycle regulatory factors had particularly high statistical significance (*P* = 3.1E-10), and included most prominently, eighteen important mitotic regulatory genes (*P* = 2.6E-6; Figure 4E). qPCR validated the decreases in four of six centromeric/mitotic genes tested: *CENP-A, Birc5, Cdc20,* and *Dbf4* and also the DNA synthesis gene *Rrm1* (Additional file 5: Figure S5B). Despite significant ontological clusters, it is notable that the fold changes in genes in the clusters mentioned above were themselves very moderate by array, possibly explaining why only some expression changes were able to be validated by qPCR.

### Changes in overall histone levels with loss of *H3f3b* in mouse embryonic fibroblasts

Since ontology analysis of our expression array data indicated widespread changes in histone family genes, we examined histone family protein levels by immunoblotting (Figure 4B). Six members of the *Hist1H2a* gene family, all encoding histone H2a protein, were downregulated (data are means of both the constitutive and conditional *H3f3b* KO MEFs relative to mean of WT samples; Figure 4C) at the RNA level, so we measured H2a proteins levels finding them at most mildly decreased in *H3f3b* KO MEFs. Two histone H3 family members (*CENPA* and *Hist1H3b*) were also downregulated at the mRNA level. We observed moderate decreases in CENP-A via immunoblot (Additional file 5: Figure S5E) but we did not see consistent changes in H3 protein levels in different KO lines (Figure 4B, Immunostaining; Additional file 3: Figure S3E). Focusing on histone genes moderately upregulated in the array study from constitutive KO data (Additional file 5: Figure S5C), we saw no consistently clear changes in their protein levels (Additional file 5: Figure S5D). Regarding H3.3 protein levels, since the constitutive *H3f3b* KO MEFs lines had very low total H3.3 protein levels, *H3f3b* may have a more dominant role in H3.3 protein production in MEFs than *H3f3a* (Figure 4B and Additional file 2: Figure S2C, D). Histone H3 marks including histone 3 phosphorylation at serine 10 (H3S10P), H3K4me3 and H3K9ac were also observed as reduced in the *H3f3b* KO cells, but perhaps not to the same degree as H3.3. Interestingly, at the protein level, all analyzed KO lines exhibited relative hyper-phosphorylation of H3.3 at Serine 31 perhaps in compensation to more pronounced reductions in total H3.3 protein (Additional file 2: Figure S2C, E).

### *H3f3b* knockout cells exhibit abnormal nuclear architecture with larger, more rounded nuclei

We analyzed images of DAPI-stained nuclei from WT1 and KO1 cell lines using the R package EBImage (http://www.bioconductor.org/). MEFs lacking *H3f3b* have abnormal nuclei in terms of both volume and shape (Figure 4G and Additional file 3: Figure S3B). *H3f3b* KO MEFs exhibit a greater than 40% mean increase in nuclear volume (Figure 4G), but a more compact morphology as measured by shape factor suggesting that loss of *H3f3b* may affect nuclear architecture/chromatin structure overall. We repeated this analysis using WT46 and WT48 compared to KO49 and KO52 and observed the same trends in nuclear volume and shape (data not shown). These global alterations in nuclear structure are also associated with brighter DAPI staining evident in the KO nuclei. The large nuclear size of some KO MEFs also was consistent with a subpopulation endocycling (Figures 3A, 4G, and Additional file 3: Figure S3B, C) [41].

### Loss of *H3f3b* alters cell cycling with increased G2/M phase

To examine cell cycle profiles more directly, cell lines WT46, WT48 and KO49, KO52 were analyzed by flow cytometry for DNA content (Figure 4H,I). *H3f3b* KO cells exhibited a nearly 40% increase in G2/M cells (*P* value <0.01), increasing from 20% to 28%. The G0/G1 phase fraction was decreased from 54% to 42% of the population. While fifteen critical DNA synthesis regulatory genes, including all the main genes of the minichromosome maintenance complex (MCM), were downregulated as assessed by array (Figure 4F), no significant change in the percent of cells in S phase was evident in the KO (Figure 4I) and the apparent changes in expression of each MCM gene by array were very small. Together, the decrease in G0/G1 cells, along with no change in S phase levels and the pronounced increase in G2/M cells support a model in which KO cells are at times blocked in M. The high frequency of chromosomal bridges in the KO cells complicates comparative (WT versus KO) measurement of mitotic cells by flow cytometry, as cells with mitotic bridges are likely to be destroyed during the process. As a result, we speculate that the *H3f3b* KO mitotic arrest is even more pronounced than evident in the flow cytometry data in Figure 4I.

### Moderate loss of genomic H3K4me3 and H3K9ac peaks in *H3f3b* knockouts as measured by chromatin immunoprecipitation-sequencing

**Figure 5:**
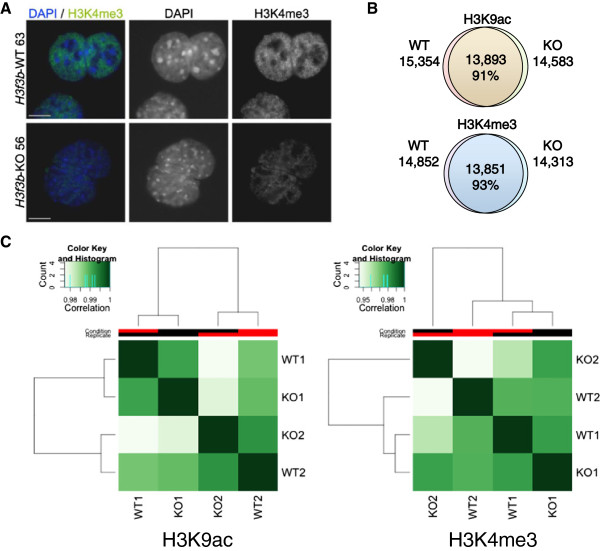
**Global changes in genomic levels of H3K9ac and H3K4me3 with loss of*H3f3b*suggest a moderate role for H3.3 in euchromatin.** (**A**) Immunostaining in wildtype (WT) 63 versus knockout (KO) 56 indicates a modest decrease in global histone 3 lysine 4 tri-methylation (H3K4me3) levels in KO nuclei. Scale bar = 10 um. (**B**) ChIP-seq analysis of H3K4me3 and histone 3 lysine 9 acetylation (H3K9ac) marks was performed, normalizing samples to input for peak calling. Venn diagrams show the number of total and overlapping peaks in WT and KO samples. Note, that for each condition the data presented are total peak numbers from merged biological duplicates of WT1 and WT2 versus KO1 and KO2. The number and percentage of overlapping peaks are indicated in the middle of each diagram. (**C**) Binding affinity clustering heatmap of H3K9ac (left) and H3K4me3 (right) peaks in two biological replicates of WT and KO MEFs. Correlation color codes are shown above each plot. Clustering was performed using R package DiffBind, which clusters the samples based on the normalized read counts for each sample at each putative histone mark peak.

To examine potential regulation of euchromatic states more generally by H3.3, we immunostained WT and *H3f3b* KO MEFs for H3K4me3, a classic euchromatic mark. We observed a modest, but consistent decrease in H3K4me3 staining in KO MEFs (Figure 5A). To more rigorously measure the potential functions of *H3f3b* and H3.3 in regulating mammalian chromatin, we conducted ChIP-Seq in WT and *H3f3b* KO MEFs for two euchromatic histone H3 marks, H3K9ac and H3K4me3 (Figure 5B). For each condition we sequenced biological duplicates and genotype-matched input controls (Additional file 10: Table S5). We aligned them to the mouse genome mm9 with Bowtie. We used two different peak callers, Sole Search (http://korflab.ucdavis.edu/software.html) and PeakRanger (http://ranger.sourceforge.net), which yielded very similar results. Sole Search peaks from biological duplicates had 95 to 98% overlap (Additional file 11: Figure S6A). When biological duplicates were merged, ChIP-Seq peaks for each condition revealed a high degree of overlap between WT and KO profiles of H3K9ac (91% of WT peaks were present in KO) and H3K4me3 (93% of WT peaks were present in KO) (Figure 5B). We used the R package DiffBind (http://www.bioconductor.org) to perform affinity clustering analysis of PeakRanger peak sets, in order to cluster the ChIP-seq samples based on tag density (Figure 5C). We observed very high correlation between all samples for each histone mark (correlation >0.9). For both H3K9ac and H3K4me3, KO and WT duplicate samples in each case failed to cluster together, indicating few reproducible differences in histone modification patterns between KO and WT.

DiffBind was then used to perform edgeR differential binding analysis. Surprisingly few H3K9ac peaks were identified by DiffBind as significantly different (FDR <0.1) between KO and WT; only one peak increased and five peaks decreased for H3K9ac. More changes were observed for H3K4me3 with 105 peaks increased and 203 peaks decreased for H3K4me3 (Additional file 12: Table S6). We used the peak2gene tool at Galaxy Cistrome (http://cistrome.org/ap/) to identify genes within 30 kb of significantly changed peaks (Additional file 12: Table S6). For both histone marks, the *H3f3b* locus itself was among the most significantly reduced peaks, which is expected due to the absence of reads mapping to that locus in KO samples. Gene ontology analysis of genes near differentially modified H3K4me3 peaks reveals that peaks reduced in KO are preferentially located near genes related to extracellular matrix (enrichment score = 6.3), while peaks increased in KO are preferentially located near genes related to protein kinases (enrichment score = 4.0) (Additional file 13: Table S7).

In agreement with the H3K4me3 Western blot data (Figure 4B), twice as many H3K4me3 peaks were significantly reduced as were increased by loss of *H3f3b*, suggesting a possible reduction in euchromatin. However, no statistically significant differences in peak heights or widths were observed consistently between WT and KO samples (Additional file 10: Table S5), nor was any consistent loss of H3K4me3 or H3K9ac observed in the vicinity of transcription start sites (TSS) in genic regions (Additional file 11: Figure S6B). H3K4me3 peaks gained and lost in the *H3f3b* KO cells were mapped relative to their distance from centromeres (Additional file 14: Figure S7), discerning no specific pattern spatially relative to centromeres.

We compared unique H3K9ac and H3K4me3 peaks from WT and KO MEFs to an existing data set of CTCF peaks in MEFs to determine whether changes in histone marks could be specifically occurring near CTCF-associated chromatin boundaries [42]. We examined two sets of peaks from our data: the peaks that overlap between WT and KO (shared peaks), and peaks that do not overlap between WT and KO (unique peaks). We did not find a correlation between unique peaks and CTCF binding sites. For H3K4me3, 42% of shared peaks and 40% of unique peaks are within 1000 bp of a CTCF peak (*P* = 0.042). For H3K9ac, 41% of shared peaks and 40% of unique peaks are within 1000 bp of a CTCF peak (*P* = 0.16). Unique peaks are therefore not preferentially found near insulator boundaries marked by CTCF binding, suggesting no major connection of H3.3 with insulator function (data not shown).

## Discussion

Disruption of *H3f3b*, one of the two H3.3-encoding genes in mice, lowers H3.3 protein levels sufficiently to induce developmental, cellular, transcriptional and chromatin-related phenotypes. Our studies suggest that one predominant developmental function of histone variant H3.3 is facilitating chromosome segregation at least in part traceable to delineation of appropriate pericentric heterochromatin and CENP-A-bound chromatin domains. Our results are in line with a previously published study in which loss of the critical K27 residue of murine H3.3 ultimately results in chromosome segregation defects and disruption in pericentric chromatin [43].

H3.3 interacts directly with DAXX, and in complex with ATRX [21]. DAXX was shown to be the primary chaperone involved in the incorporation of epitope-tagged H3.3 protein into MEF chromatin, and both DAXX and ATRX are found to be strongly enriched at pericentric DNA repeats [22]. Removal of ATRX, which targets DAXX to the pericentric region, disrupts pericentric heterochromatin composition in oocytes, at least partially because of a failure to recruit DAXX to these genomic regions. This ultimately leads to increased incidences of aneuploidy and chromosome segregation defects [44].

Pericentric DNA is transcribed during S-phase and during mitosis in mouse cells [45], and the regulation of proper amounts of pericentric transcript has been implicated by numerous studies to be involved in both proper pericentric heterochromatin formation (reviewed in [46]) and in appropriate chromosome segregation [43,47]. Pericentric DNA transcription was found to be altered when critical H3.3 residues were changed [43], and upon knockdown of H3.3 by siRNA or loss of DAXX [22]. These published findings combined with our data here, lead us to hypothesize that deposition of H3.3 plays a role in the regulation of pericentric heterochromatin.

Targeted disruption of *H3f3b* and the resulting decrease in H3.3 protein lead to a spectrum of severe karyotypic abnormalities, an increase of cells in G2/M-phase of the cell cycle, and an increased incidence of chromosomal bridges. We theorize that these specific phenotypic changes are linked. One model explaining these observations invokes a connection between the proper amounts of H3.3 protein in pericentric heterochromatin and the generation of appropriate levels of pericentric transcript. Disruption of either component could lead to defects in chromosome segregation and chromatid cohesion during cell division [47]. In this regard, future studies will need to address pericentric transcript levels in the *H3f3b* KO MEF lines examined here.

Our data also demonstrate an increase in pericentric heterochromatin, kinetochore, and CENP-A foci in interphase nuclei. One published model for H3.3 function describes H3.3 acting in a gap-filling manner following transcription, possibly in part to protect DNA [13,20]. Following H3.3 loss in *Drosophila*, H3.3 chaperones ATRX/DAXX were found to maintain association in place of H3.3 in regions normally bound by this histone variant [13]. Thus, another possible explanation for the observed karyotypic and chromosome segregation defects may be the aberrant deposition of other factors involved in kinetochore function, such as CENP-A, in regions normally bound by H3.3 that may affect the organization of proper centromere or heterochromatin domains. Supporting this finding, H3.3 was found to be deposited in centromeric chromatin as a placeholder for CENP-A [9]. In addition, H3.3 was shown to inhibit the spread of heterochromatin domains in *Drosophila*[48]. The aberrant spread of factors, such as CENP-A, outside of their normal chromatin domains as observed by staining could also potentially result in the observed increased rates of chromosome/chromatid fragments and breaks, cell cycle defects, and chromosomal bridges, leading to tri-radial or rearranged chromosomes. Although we were not able to detect an increase in CENP-A protein expression by Western blot on whole MEF cell extracts, this may be due to differences in sensitivity or antigen availability between staining (showing nuclear CENP-A) and blotting (whole cell CENP-A) methods.

It is formally possible that the observed increases in pericentric heterochromatin, kinetochore, and CENP-A foci could be due to increased DNA content within the *H3f3b* KO MEFs. Although we did not observe increased rates of polyploidy in *H3f3b* KO MEFs when compared to WT, it is possible that our karyotyping analysis may have missed such events that ultimately resulted in cell death, thereby removing these MEFs from the population prior to analysis. However, our findings of significant, but moderate increases in CENP-A and CREST foci are not sufficiently supported mechanistically simply by an increase in DNA content as (1) the rates of endoreduplication in our karyotyping analysis were extremely low (3%); (2) the absolute percentage of cells found to be in G2/M-phase of the cell cycle in *H3f3b* KO MEFs was only increased by 8% (though statistically significant versus WT), and (3) hundreds of MEF nuclei were analyzed for pericentric heterochromatin, CENP-A, and kinetochore foci and all of these were found to be increased in *H3f3b* KO MEF nuclei. Though we did observe an increase in nuclear size, karyotyping analysis indicates similar DNA content between each genotype of cells (Table 1); thus, one explanation for the higher nuclear volume in one of the clones could be due to the impact that loss of *H3f3b* or H3.3 protein has on chromatin and nuclear structure.

Taken together, our data supports the existing models of alternate deposition of H3.3-related factors upon H3.3 depletion. However, our data expands on the current models and suggests that upon H3.3 reduction, other factors linked to H3.3 deposition such as CENP-A may spread beyond the borders of their normal domains. This in turn may result in ectopic heterochromatin regions that alter the recruitment of kinetochore proteins or other factors involved in the generation of pericentric heterochromatin domains.

H3.3 has been associated previously with transcriptionally active chromatin domains [1,2], and our data indicate that loss of H3.3 changes the transcriptome, but only moderately. Consistent with this notion, ChIP-Seq studies in mouse ES cells indicated that tagged H3.3 was associated not only with actively transcribed genes, but also with repressed and poised genes [3]. While genic H3.3 deposition is strictly HIRA-dependent in ES cells, such H3.3 enrichment is not required for maintenance of the ES cell transcriptome [3]. Alternatively, H3.3 may have a more major role in transcriptome regulation than apparent in our knockout studies because there could also be compensatory mechanisms at work that attenuate the measurable effects of decreased H3.3 levels on the transcriptome. Since total H3 protein levels are not consistently elevated in the *H3f3b* KO cells (a phenomenon observed in the fly with loss of H3.3), it seems most likely that such compensation would occur through changes in canonical H3 PTMs to make them more similar to those in H3.3.

It is also possible that although total H3.3 protein levels in *H3f3b* KO MEFs were substantially reduced, we still did not lower total H3.3 levels far enough (because of the remaining *H3f3a* gene) to strongly impact global promoter chromatin or transcriptional activity. Such a scenario would depend on the minimum levels of H3.3 protein required to fulfill these transcription-related functions being far lower than its other functions (for example, chromosome segregation) that were strongly perturbed in the *H3f3b* knockout. Alternatively in the context of loss of *H3f3b*, remaining H3.3 could be preferentially maintained near TSSs (where we saw no major change in H3K4me3 by ChIP-Seq). The ontology of the genes that did exhibit changes in expression in the KO cells was nonetheless quite interesting. Such changes suggest key links between H3.3 and core regulatory machinery of cell cycle (in particular mitosis and DNA synthesis) and chromatin. Another interesting possibility is that CENP-A, as a histone H3 family member, compensated at a transcriptional level to some extent for the loss of H3.3 by in essence filling in for H3.3 at TSS regions, which could also explain the apparent ectopic regions of CENP-A staining. Future ChIP-Seq studies for CENP-A in H3.3 loss-of-function cells will aid in exploring this possibility.

The links between H3.3 mutations and glioblastoma [31-33] as well as to cellular reprogramming [7] are important emerging areas of study. At this time, the functional consequences of the specific K27 and G34 mutations in histone H3.3 remain unknown. Given that deletions in H3.3 have not to date been observed in tumors, one might speculate that the H3.3 point mutations in glioblastoma are gain-of-function; however, there is no direct evidence to this effect. Alternatively, the H3.3 mutations could be either loss-of-function or change-of-function. The coincident mutations in the H3.3 chaperones DAXX and ATRX might also suggest altered function of the H3.3 pathway more generally in these tumors. Our data reported in the current knockout study suggest possible insights into mechanisms by which changes in H3.3 function might contribute to tumorigenesis. Loss of H3.3 compromised genome integrity, altered chromosome segregation, and changed both the transcriptome and epigenome. Each of these alterations could be modeled to contribute to cancer formation.

Future functional genomics and biochemical studies will likely further clarify the role of histone variant H3.3 in normal and neoplastic cellular functions. Production of mammalian cells completely lacking all H3.3 protein, assuming such cells can survive at all (as they do in the fly) [16,24], will also provide valuable tools for further interrogating H3.3 genomic and biological functions.

## Conclusions

Here we report that histone variant H3.3 is required for normal murine development as targeted disruption of the H3.3-encoding gene *H3f3b* leads to numerous defects and a semi-lethal phenotype in mice. Defects in *H3f3b* KO cells include abnormal chromosome segregation and ectopic CENP-A domains. In addition, loss of *H3f3b* and lowering of H3.3 levels induced changes more generally consistent with spreading of heterochromatin. KOs also displayed abnormal cell cycling with cells accumulating in mitosis. Karyotypic abnormalities including endoreduplication were observed in KO cells as well. Additionally, *H3f3b* KO cells exhibited changes in both the transcriptome and epigenome. The epigenetic changes in KOs in H3K9ac and H3K4me3 peaks were, however, not evident specifically near TSSs or centromeres. Overall, these studies provide novel and important insights into endogenous H3.3 function in development, transcription, epigenetic regulation, and chromosome segregation.

## Methods

Additional methodological details can be found in the Additional file 15.

All animal studies were approved by the University of California-Davis Institutional Animal Care and Use Committee (IACUC).

### Mouse production and genotyping

*H3f3b* targeted mES cells (JM8.F6) were produced by electroporation of the targeting vector (Figure 1A) and selection with G418. Single copy presence of the recombined allele was verified by genomic PCR. Targeted ES cells were injected into BALB/c blastocysts. Chimeric mice were bred with WT and germline transmission judged by coat color and genomic PCR. All genotyping was performed using Bioneer AccuPower Pre-mix PCR tubes (http://www.bioneer.com/), which require only water, DNA and primerPrimers used (refer to Figure 1A for diagram of their relative locations indicated by arrows):

1. KO Primer: GCTCGACTAGAGCTTGCGGAAC

2. FL Primer: CTGATGGCGAGCTCAGACCATAAC

3. Common: CAGTTTGAGTGCCTGAGGACAAG

4. WT Primer: GAACTCAGGACCTTAGGATGAGCAGT

### Chromatin immunoprecipitation-sequencing (ChIP-Seq)

Chromatin immunoprecipitation was carried out on WT1, WT2 and KO1, KO2 MEFs as described [49]. For more detail see Additional file 15.

### Acid extracts

Acid extracts were prepared from biological replicates of *H3f3b*^Δ/Δ^ and *H3f3b*^WT/WT^ MEFs according to the Abcam protocol. Cells were harvested and washed twice with ice-cold PBS supplemented with 5mM sodium butyrate. Cells were then incubated in triton extraction buffer (TEB: 0.5% Triton X-100, 2mM PMSF, 0.02% sodium azide) for 10 minutes at 4°C and pelleted. After another TEB wash, histones were extracted in 0.2 N HCl overnight at 4°C. Cells were pelleted, and supernatant was saved and aliquotted for immunoblotting. All extracts were neutralized with NaOH prior to use.

### Mouse embryonic fibroblasts isolation

*H3f3b*^Δ/WT^ mice were crossed, and embryos were isolated at E12.5. The head and visceral organs were removed and remaining tissue was trypsinized and disassociated. Trypsin was then neutralized and remaining cells were cultured with MEF medium (10% FBS, 1% non-essential amino acids, 1% Glutamine, DMEM). Tissue was removed from each embryo head to be used for genotyping.

*H3f3b*^Fl/Fl^ MEFs were prepared in a similar manner from E12.5 embryos obtained by *H3f3b*^Fl/Fl^ by *H3f3b*^Fl/WT^ crosses.

### Antibodies

A list of all antibodies used in this study can be found in Additional file 16: Table S8.

### Immunocytochemistry

WT and KO E12.5 MEFs were plated on gelatin coated glass cover slips in each well of a six-well plate. After a day of growth, cells were washed once with 1x PBS, and fixed for 15 minutes with 4% paraformaldehyde. Cells were then washed again, and blocked (5% BSA, 3% normal goat serum, 0.3% Triton X-100 in PBS) for one hour at room temperature (RT) Primary antibodies were added in carrier solution (3% normal goat serum, 0.3% Triton X-100 in PBS) and incubated overnight at 4°C. Cells were then washed 3×5 min in PBS, then incubated in carrier solution with AlexaFluor 546 or 488 anti-rabbit secondary IgG. The cover slips were washed four times for 5 minutes each time with PBS and mounted on slides with Vectashield mounting medium with DAPI (Vector Labs- http://www.vectorlabs.com/).

To arrest the MEFs, cells were treated with colcemid (demecolcine) at 0.15 ug/mL. Slides were incubated with the drug for 4 to 5 hours. For specific antibody dilutions see Additional file 16: Table S8.

### Western blots

Equivalent levels of protein were run through 6 to 12% Bis-Tris gel as indicated by Invitrogen protocol (http://www.invitrogen.com/site/us/en/home.html). Protein was then transferred onto polyvinylidene difluoride (PVDF) membrane and blocked in 5% milk. Two methods were employed when incubating western blots with primary antibodies. Depending on the antibody, membranes were either 1) blocked one hour in 5% milk/TBST at RT and then incubated in primary overnight at 4°C, or 2) blocked overnight at 4°C in 5% milk/TBST then incubated with primary for two hours at RT. All secondary antibodies were applied for 1 hour at RT.

### Q-PCR analysis

The TaqMan probe for *H3f3b, H3f3a,* and the control housekeeping gene, *Eif4g2*, were obtained from Applied Biosystems (http://www.invitrogen.com/site/us/en/home/brands/taqman.html). Each reaction utilized 10 ng of cDNA, and separate reactions containing 10 ng of RNA were used to check for genomic DNA contamination. Samples were prepared according to Applied Biosystems protocol, and run on Roche LightCycler 480 with included software (https://www.roche-applied-science.com/sis/rtpcr/htc/index.jsp). Results were analyzed using the 2^-δδCt^ method. All other genes were analyzed using Absolute Blue QPCR Master Mix (http://www.thermoscientific.com/ecomm/servlet/productsdetail?productId=11956387) for SYBR green-based quantitative PCR (qPCR). Fold changes were normalized to *PPIA* and analyzed by the 2^-δδCt^ method. For list of primers used, see Additional file 15.

### Gene expression microarray analysis

RNA from constitutive *H3f3b* KO 1 and KO 2 and WT 1 and WT 2 MEFs was submitted in biological duplicates. Conditional knockout samples were submitted in analytical duplicates. A total of 500 ng of RNA from each sample was used on an Illumina Sentrix Mouse Ref-8 Expression BeadChip (http://www.illumina.com/products/mouseref-8_v2_expression_beadchip_kit.ilmn) containing over 25,000 probes. Amplification and hybridization were performed at the University of California-Davis expression analysis core according to the Illumina protocol. Microarray data was analyzed using GenomeStudio version 3.1.1.0 (http://www.illumina.com/software/genomestudio_software.ilmn).

### Cell cycle analysis

The 10 cm plates of KO 49 and 52 and WT 46 and 48 MEFs were harvested at approximately 70% confluency. Cells were incubated in a hypotonic lysis/staining solution (0.1% sodium citrate, 0.1% Triton X-100, 10 μg/mL propidium iodide, 100 μg/mL RNase A) for 1 to 3 hours at RT, and then nuclei were analyzed by flow cytometry on a Dako Cyan ADP. Results were quantified using Cylchred software (Terry Hoy, Cardiff University; available upon request).

### Kinetochore foci and pericentric heterochromatin foci expression in mouse embryonic fibroblasts

*H3f3b* WT (n = 94, two clones) and *H3f3b* KO (n = 81, two clones) nuclei were stained with CREST serum for total kinetochore protein expression and with antibodies recognizing the H3 variant and kinetochore component CENP-A. An average of 17 confocal sections per nuclei was analyzed using ImageJ software (http://rsb.info.nih.gov/ij/) for CREST, CENP-A, and DAPI-positive foci. To verify our data, we also analyzed a separate set of *H3f3b* WT and KO nuclei under identical conditions (n = 91, two *H3f3b* WT clones; n = 86, two *H3f3b* KO clones).

### Telomere fluorescent *in-situ* hybridization

These studies were conducted largely as described [50].

### Data access

ChIP-Seq and Expression Array data sets have accession numbers [NCBI GEO:GSE34546] and [NCBI-GEO:GSE35321], respectively.

## Abbreviations

ChIP-Seq: Chromatin immunoprecipitation-sequencing; ES: Embryonic stem cells; FISH: Fluorescent *in-situ* hybridization; FL: Floxed; H3K4me3: Histone H3 lysine 4 tri-methylation; H3K9ac: Histone H3 lysine 9 acetylation; H3.3S31P: Histone H3.3 phosphorylated at serine 31; ICC-IC: Immunocytochemistry-Immunofluorescence; KO: Knockout; MCM: Minichromosome maintenance complex; MEF: Mouse embryonic fibroblasts; MFI: Mean fluorescence intensity; MSC: Mesenchymal stem cell; PML: Promyelocytic leukemia; PTM: Posttranslational modification; RD: Replication dependent; RI: Replication independent; RT: Room temperature; TSS: Transcription start site; WT: Wildtype.

## Competing interests

The authors declare that they have no competing interests.

## Authors’ contributions

KB conducted all mouse breeding and genotyping to produce the H3f3b knockout strain. KB also did the majority of experiments conducted that are reported in this manuscript. BY conducted most of the immunostaining studies of MEFs, did the kinetochore staining and quantitative analysis, and aided in data interpretation. BB conducted data analysis from the ChIP-Seq studies and conducted other experiments including cell cycle and cell shape analysis, JR aided in expression microarray analysis, HO conducted the ChIP-Seq sequencing and aided in data analysis, RC prepared ChIPs and aided in the ChIP-Seq studies, and PK conceived of the project, supervised the studies, did experimental design and data interpretation, and together with KB did the majority of the writing of the manuscript. All authors read, helped write and edit, and have approved the manuscript.

## Supplementary Material

Additional file1: Figure S1 (**A**) Genomic location, (**B**) genotyping genomic PCR, and (**C**) southern blotting assays/probes for *H3f3b.* Click here for file

Additional file 2: Figure S2 (**A**-**B**) H3f3a mRNA levels in (**A**) other knockout (KO) mouse embryonic fibroblasts (MEF) lines produced and (**B**) KO embryonic tissue. (**C**) H3.3 protein levels of total H3.3 protein levels in other MEF lines produced. (**D**) Western quantification of H3.3 protein levels in KO 1, 2, 49 and 52. (**E**) Western quantification of H3.3S31P protein levels in KO1, 2, 49 and 52 relative to H3.3 protein levels. Click here for file

Additional file 3: Figure S3 (**A**) (top) Example of normal wildtype (WT) karyotype. (Bottom) Example of knockout 1 (KO1) karyotype exhibiting breaks. (**B**) Abnormal shape factor and evidence of elevated levels of endoreduplication in the KO, DAPI staining and quantitation. Arrows indicate nuclei with possible endoreduplication. (**C**) Metaphase spread of mouse embryonic fibroblasts (MEF) line KO2 show endoreduplication. (**D**) DAPI/CREST staining for mouse acrocentric chromosomes and centromeric regions in WT 63 and KO 56. Scale bar = 10 um. (**E**) Immunostaining for total H3 in WT 46 and KO 49 (MEFs). Scale bar = 20 um. Click here for file

Additional file 4: Figure S4 Telomere fluorescent *in-situ* hybridization (FISH) (green) on wildtype (WT) and H3f3b knockout (KO) cells either arrested in (**i**) metaphase or (**ii**) unsynchronized in interphase. Click here for file

Additional file 5: Figure S5 (**A**) Total RNA levels in wildtype (WT) and knockout (KO) mouse embryonic fibroblasts (MEFs). (**B**) qPCR validation of genes downregulated on microarray. (**C**) Histone genes with upregulated expression in constitutive KO – array data. (**D**) Immunoblots of histone H2b and H4. (**E**) Western blot and associated quantification of CENP-A protein in KO 49 and 52 versus mean WT. Click here for file

Additional file 6: Table S1 Gene expression analysis on constitutive and conditional mouse embryonic fibroblasts (MEFs) for genes that were up- or downregulated by at least 2 fold. (*P* <0.05). Click here for file

Additional file 7: Table S2 Gene expression analysis on constitutive and conditional mouse embryonic fibroblasts (MEFs) for genes that were up- or downregulated by at least 1.5 fold. Click here for file

Additional file 8: Table S3 Ontological expression analysis on constitutive mouse embryonic fibroblasts (MEFs) for genes upregulated. Click here for file

Additional file 9: Table S4 Ontological expression analysis on constitutive mouse embryonic fibroblasts (MEFs) for genes downregulated. Click here for file

Additional file 10: Table S5 Basic statistics from the alignment and peak calling portion of the chromatin immunoprecipitation-sequencing (ChIP-Seq) analysis. Sequencing reads were aligned using Bowtie, followed by peak calling using both PeakRanger and Sole Search. Click here for file

Additional file 11: Figure S6 (**A**) Chromatin immunoprecipitation-sequencing (ChIP-Seq) global peak overlap between two biological replicates for each condition. (**B**) Average profile of ChIP-Seq peaks in the region from -1000 to +1000 bp around the transcription start site (TSS) of genes. Click here for file

Additional file 12: Table S6 Chromatin immunoprecipitation-sequencing (ChIP-Seq) differential peak analysis. Differential H3K4me3 and H3K9ac peaks were identified using R package DiffBind with False Discovery Rate < 0.1. Peak annotations within 30 kb of each peak were generated using Galaxy Cistrome tool peak2gene. Click here for file

Additional file 13: Table S7 Ontology of genes within 30 kb of H3K4me3 peaks that are significantly different in knockout (KO) mouse embryonic fibroblasts (MEFs) 1 and 2 versus wildtype (WT). Click here for file

Additional file 14: Figure S7 Histogram of H3K4me3 peaks that are significantly reduced (top) or increased (bottom) in the knockout (KO) compared to wildtype (WT), based on two biological replicates for each genotype. Histograms are divided into one plot for each chromosome. Chromosomal location is shown on the x-axis, scaled from 0 (centromere) to 1 (telomere); the y-axis shows fold change of significantly changed peaks (FDR <0.1). Graphs were produced using the R package ggplot2. Click here for file

Additional file 15 Supplemental Methods. Click here for file

Additional file 16: Table S8 Antibody list. Click here for file
