# Supplementary material for: Endogenous mammalian histone H3.3 exhibits chromatin-related functions during development
Source: Epigenetics Chromatin. 2013 Apr 9;6:7. doi: 10.1186/1756-8935-6-7 (PMC3635903; doi:10.1186/1756-8935-6-7)
Supplement: Additional file1: Figure S1 — (A) Genomic location, (B) genotyping genomic PCR, and (C) southern blotting assays/probes for H3f3b. [file 1756-8935-6-7-S1.ppt]

## Slide 1
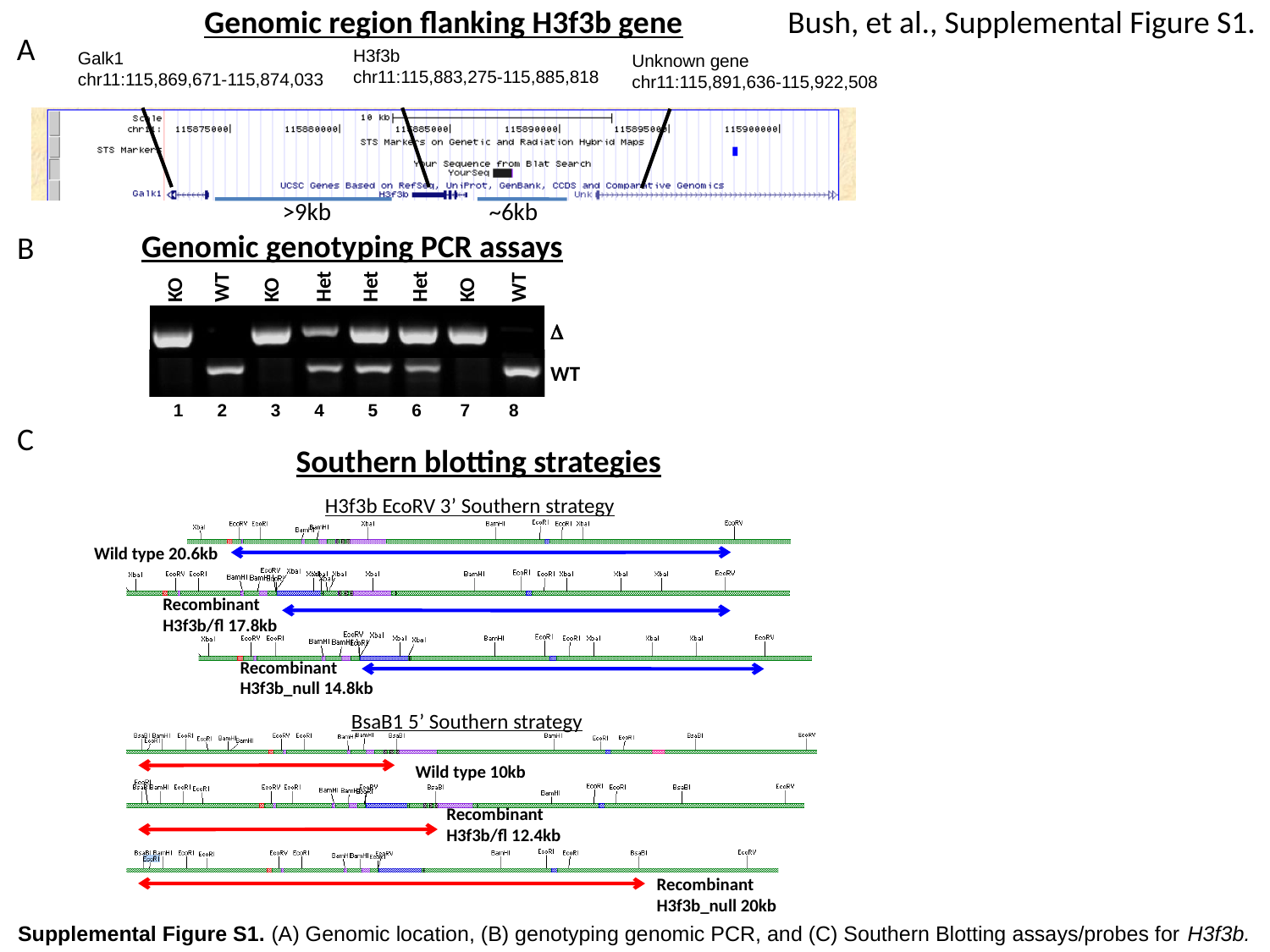

Genomic region flanking H3f3b gene
Bush, et al., Supplemental Figure S1.
A
H3f3b
chr11:115,883,275-115,885,818
Galk1
chr11:115,869,671-115,874,033
Unknown gene
chr11:115,891,636-115,922,508
>9kb
~6kb
Genomic genotyping PCR assays
B
WT
Het
Het
Het
WT
KO
KO
KO
Δ
WT
 1 2 3 4 5 6 7 8
C
Southern blotting strategies
H3f3b EcoRV 3’ Southern strategy
Wild type 20.6kb
Recombinant
H3f3b/fl 17.8kb
Recombinant
H3f3b_null 14.8kb
BsaB1 5’ Southern strategy
Wild type 10kb
Recombinant
H3f3b/fl 12.4kb
Recombinant
H3f3b_null 20kb
Supplemental Figure S1. (A) Genomic location, (B) genotyping genomic PCR, and (C) Southern Blotting assays/probes for H3f3b.
