# Supplementary material for: Endogenous mammalian histone H3.3 exhibits chromatin-related functions during development
Source: Epigenetics Chromatin. 2013 Apr 9;6:7. doi: 10.1186/1756-8935-6-7 (PMC3635903; doi:10.1186/1756-8935-6-7)
Supplement: Additional file 2: Figure S2 — (A-B) H3f3a mRNA levels in (A) other knockout (KO) mouse embryonic fibroblasts (MEF) lines produced and (B) KO embryonic tissue. (C) H3.3 protein levels of total H3.3 protein levels in other MEF lines produced. (D) Western quantification of H3.3 protein levels in KO 1, 2, 49 and 52. (E) Western quantification of H3.3S31P protein levels in KO1, 2, 49 and 52 relative to H3.3 protein levels. [file 1756-8935-6-7-S2.ppt]

## Slide 1
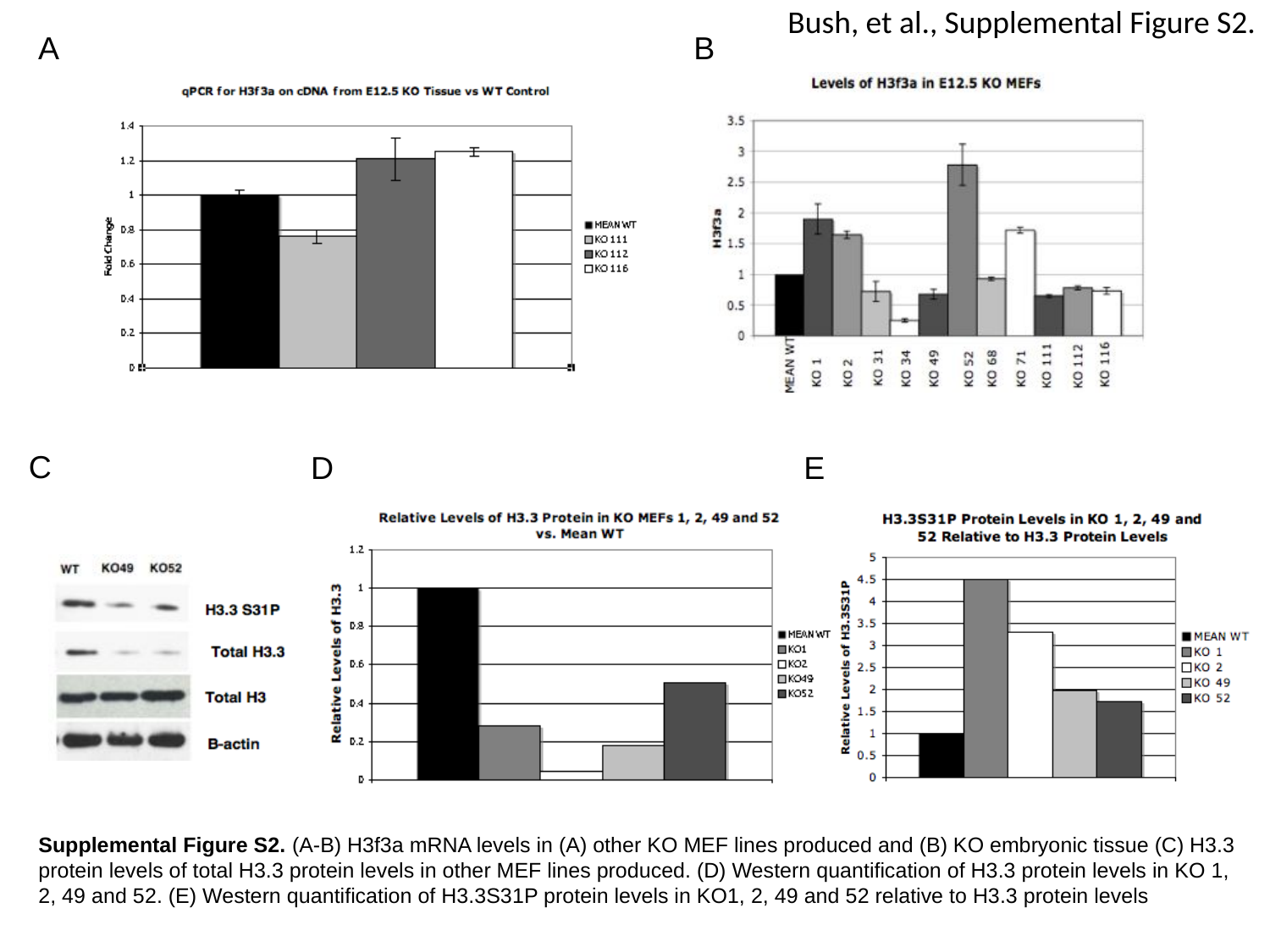

Bush, et al., Supplemental Figure S2.
A
B
C
D
E
Supplemental Figure S2. (A-B) H3f3a mRNA levels in (A) other KO MEF lines produced and (B) KO embryonic tissue (C) H3.3 protein levels of total H3.3 protein levels in other MEF lines produced. (D) Western quantification of H3.3 protein levels in KO 1, 2, 49 and 52. (E) Western quantification of H3.3S31P protein levels in KO1, 2, 49 and 52 relative to H3.3 protein levels
