# Supplementary material for: Endogenous mammalian histone H3.3 exhibits chromatin-related functions during development
Source: Epigenetics Chromatin. 2013 Apr 9;6:7. doi: 10.1186/1756-8935-6-7 (PMC3635903; doi:10.1186/1756-8935-6-7)
Supplement: Additional file 3: Figure S3 — (A) (top) Example of normal wildtype (WT) karyotype. (Bottom) Example of knockout 1 (KO1) karyotype exhibiting breaks. (B) Abnormal shape factor and evidence of elevated levels of endoreduplication in the KO, DAPI staining and quantitation. Arrows indicate nuclei with possible endoreduplication. (C) Metaphase spread of mouse embryonic fibroblasts (MEF) line KO2 show endoreduplication. (D) DAPI/CREST staining for mouse acrocentric chromosomes and centromeric regions in WT 63 and KO 56. Scale bar = 10 um. (E) Immunostaining for total H3 in WT 46 and KO 49 (MEFs). Scale bar = 20 um. [file 1756-8935-6-7-S3.ppt]

## Slide 1
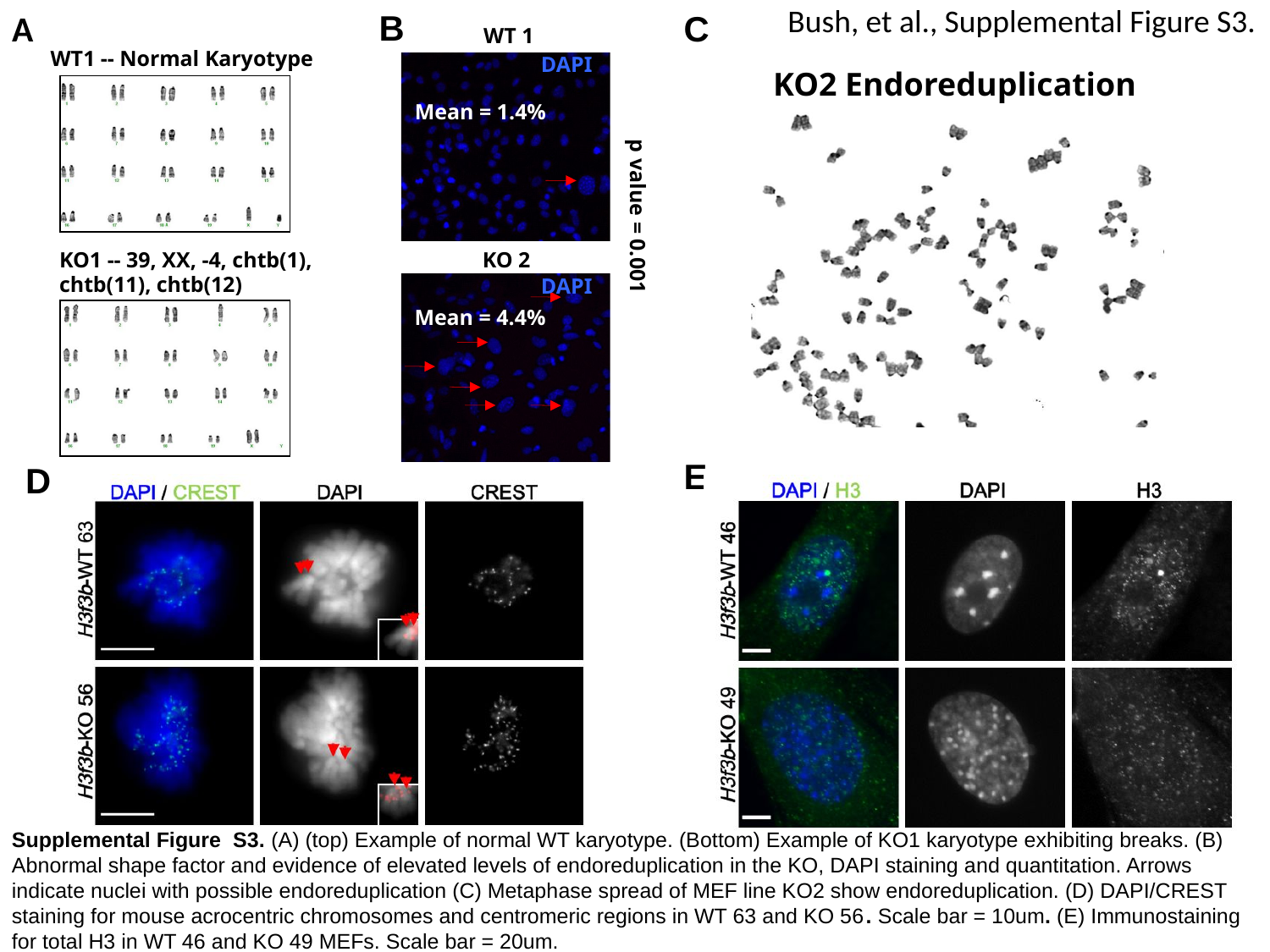

A
C
Bush, et al., Supplemental Figure S3.
B
WT 1
WT1 -- Normal Karyotype
DAPI
KO2 Endoreduplication
Mean = 1.4%
p value = 0.001
KO1 -- 39, XX, -4, chtb(1),
chtb(11), chtb(12)
KO 2
DAPI
Mean = 4.4%
E
D
Supplemental Figure S3. (A) (top) Example of normal WT karyotype. (Bottom) Example of KO1 karyotype exhibiting breaks. (B) Abnormal shape factor and evidence of elevated levels of endoreduplication in the KO, DAPI staining and quantitation. Arrows indicate nuclei with possible endoreduplication (C) Metaphase spread of MEF line KO2 show endoreduplication. (D) DAPI/CREST staining for mouse acrocentric chromosomes and centromeric regions in WT 63 and KO 56. Scale bar = 10um. (E) Immunostaining for total H3 in WT 46 and KO 49 MEFs. Scale bar = 20um.
