# Supplementary material for: Endogenous mammalian histone H3.3 exhibits chromatin-related functions during development
Source: Epigenetics Chromatin. 2013 Apr 9;6:7. doi: 10.1186/1756-8935-6-7 (PMC3635903; doi:10.1186/1756-8935-6-7)
Supplement: Additional file 4: Figure S4 — Telomere fluorescent in-situ hybridization (FISH) (green) on wildtype (WT) and H3f3b knockout (KO) cells either arrested in (i) metaphase or (ii) unsynchronized in interphase. [file 1756-8935-6-7-S4.ppt]

## Slide 1
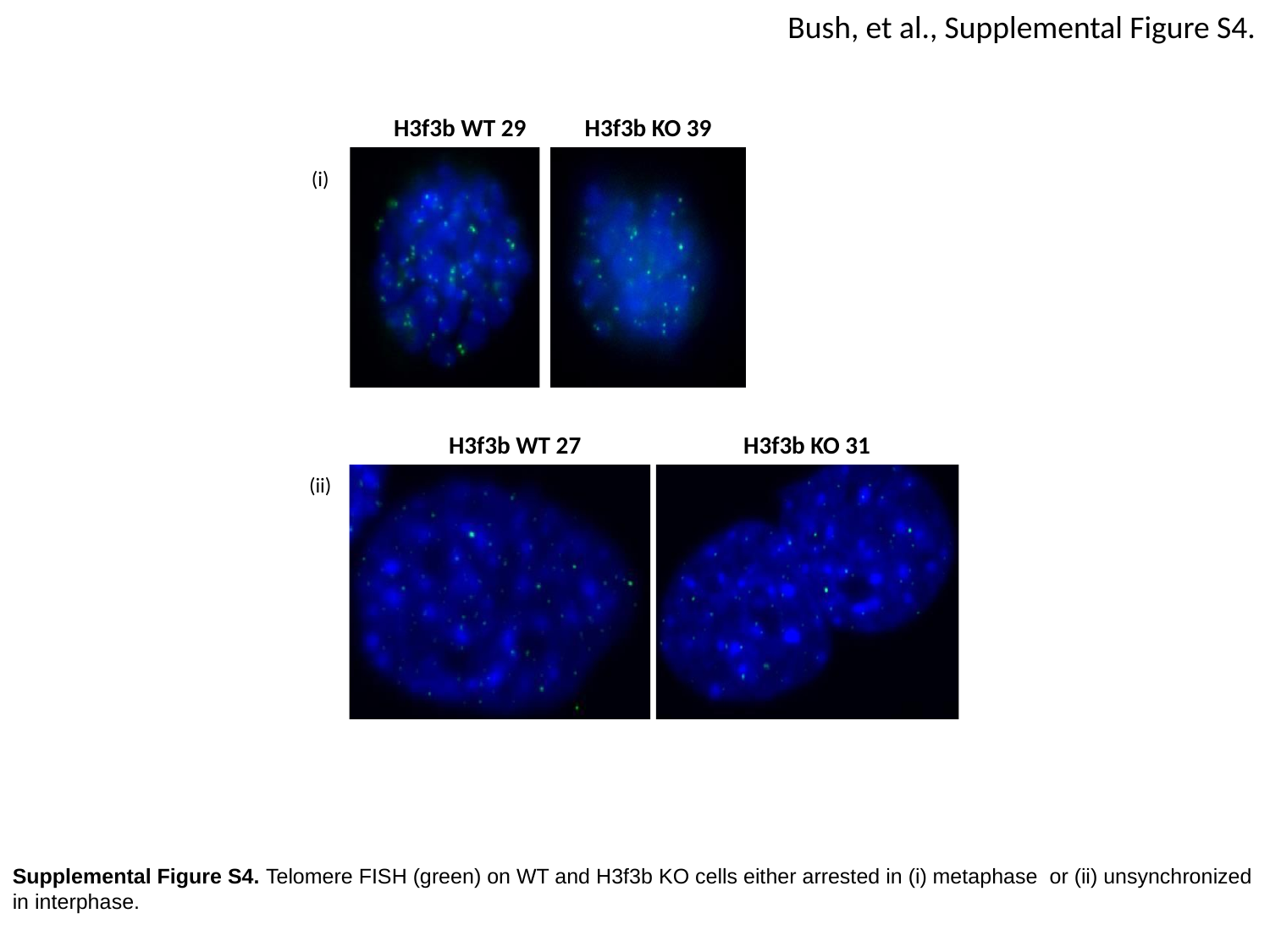

Bush, et al., Supplemental Figure S4.
H3f3b WT 29
H3f3b KO 39
(i)
H3f3b WT 27
H3f3b KO 31
(ii)
Supplemental Figure S4. Telomere FISH (green) on WT and H3f3b KO cells either arrested in (i) metaphase or (ii) unsynchronized in interphase.
