# Supplementary material for: Endogenous mammalian histone H3.3 exhibits chromatin-related functions during development
Source: Epigenetics Chromatin. 2013 Apr 9;6:7. doi: 10.1186/1756-8935-6-7 (PMC3635903; doi:10.1186/1756-8935-6-7)
Supplement: Additional file 5: Figure S5 — (A) Total RNA levels in wildtype (WT) and knockout (KO) mouse embryonic fibroblasts (MEFs). (B) qPCR validation of genes downregulated on microarray. (C) Histone genes with upregulated expression in constitutive KO – array data. (D) Immunoblots of histone H2b and H4. (E) Western blot and associated quantification of CENP-A protein in KO 49 and 52 versus mean WT. [file 1756-8935-6-7-S5.ppt]

## Slide 1
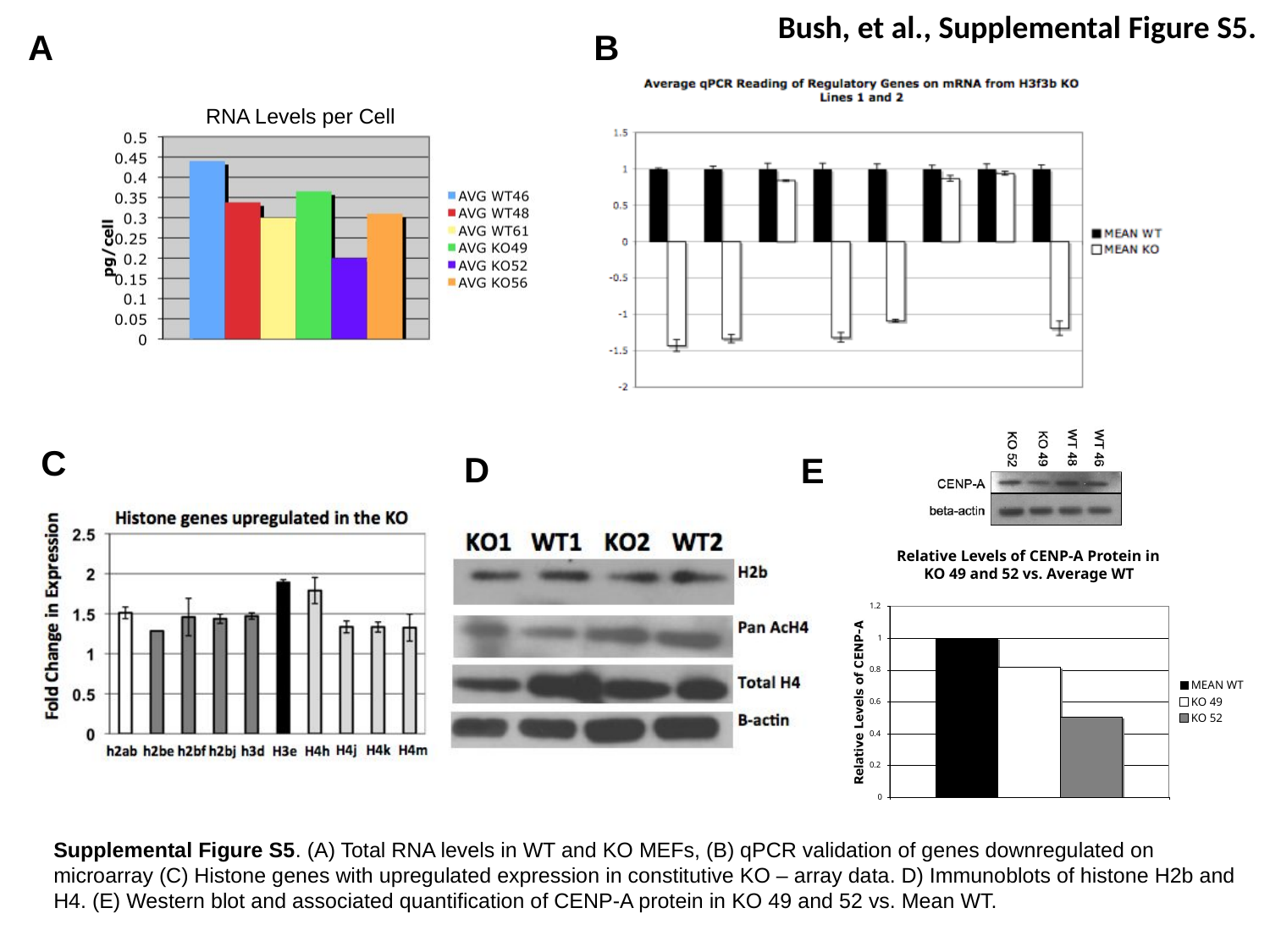

Bush, et al., Supplemental Figure S5.
A
B
RNA Levels per Cell
C
D
E
Supplemental Figure S5. (A) Total RNA levels in WT and KO MEFs, (B) qPCR validation of genes downregulated on microarray (C) Histone genes with upregulated expression in constitutive KO – array data. D) Immunoblots of histone H2b and H4. (E) Western blot and associated quantification of CENP-A protein in KO 49 and 52 vs. Mean WT.
