# Supplementary material for: Endogenous mammalian histone H3.3 exhibits chromatin-related functions during development
Source: Epigenetics Chromatin. 2013 Apr 9;6:7. doi: 10.1186/1756-8935-6-7 (PMC3635903; doi:10.1186/1756-8935-6-7)
Supplement: Additional file 11: Figure S6 — (A) Chromatin immunoprecipitation-sequencing (ChIP-Seq) global peak overlap between two biological replicates for each condition. (B) Average profile of ChIP-Seq peaks in the region from -1000 to +1000 bp around the transcription start site (TSS) of genes. [file 1756-8935-6-7-S11.ppt]

## Slide 1
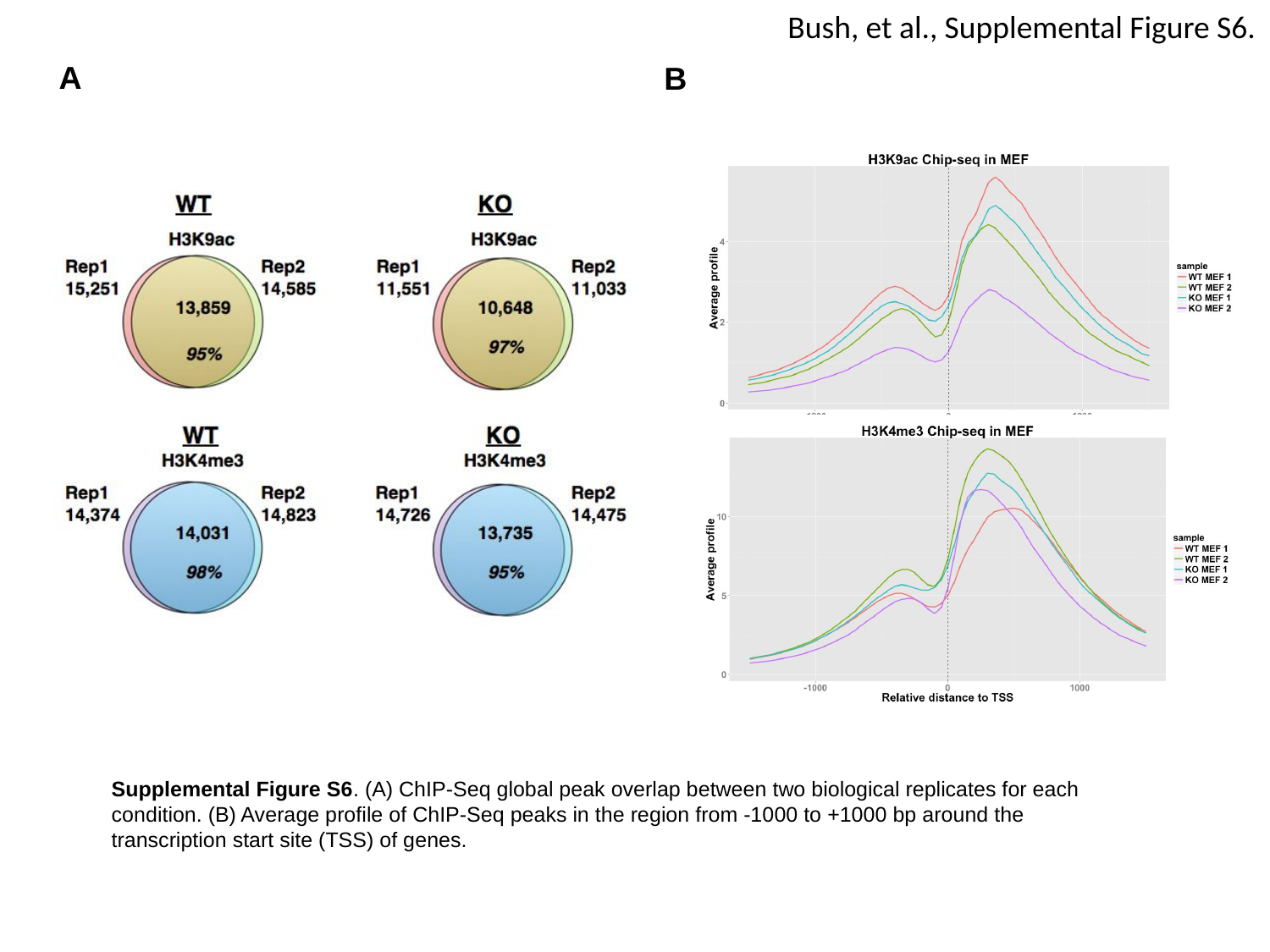

Bush, et al., Supplemental Figure S6.
A
B
Supplemental Figure S6. (A) ChIP-Seq global peak overlap between two biological replicates for each condition. (B) Average profile of ChIP-Seq peaks in the region from -1000 to +1000 bp around the transcription start site (TSS) of genes.
