# Supplementary material for: Endogenous mammalian histone H3.3 exhibits chromatin-related functions during development
Source: Epigenetics Chromatin. 2013 Apr 9;6:7. doi: 10.1186/1756-8935-6-7 (PMC3635903; doi:10.1186/1756-8935-6-7)
Supplement: Additional file 14: Figure S7 — Histogram of H3K4me3 peaks that are significantly reduced (top) or increased (bottom) in the knockout (KO) compared to wildtype (WT), based on two biological replicates for each genotype. Histograms are divided into one plot for each chromosome. Chromosomal location is shown on the x-axis, scaled from 0 (centromere) to 1 (telomere); the y-axis shows fold change of significantly changed peaks (FDR <0.1). Graphs were produced using the R package ggplot2. [file 1756-8935-6-7-S14.ppt]

## Slide 1
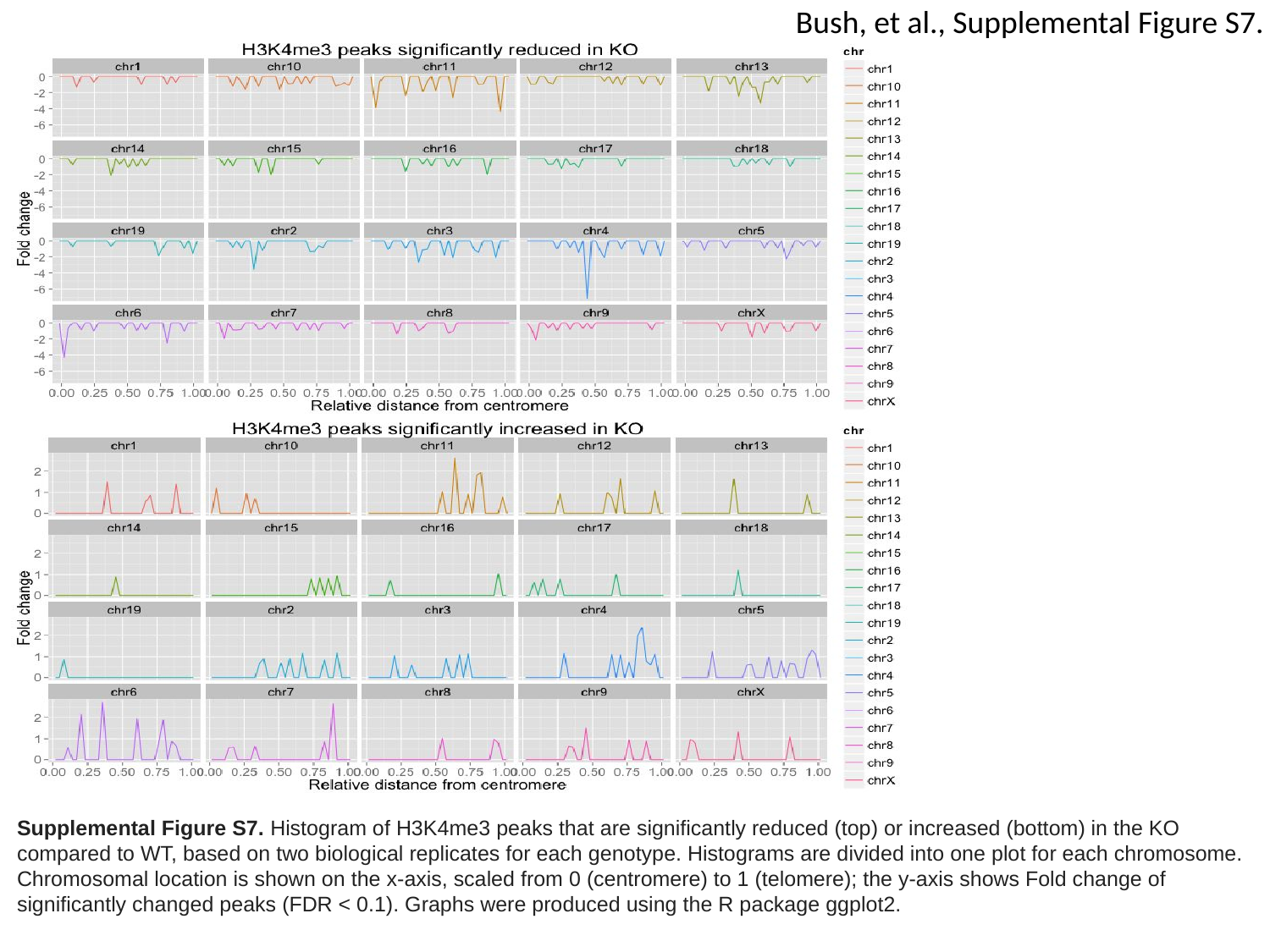

Bush, et al., Supplemental Figure S7.
Supplemental Figure S7. Histogram of H3K4me3 peaks that are significantly reduced (top) or increased (bottom) in the KO compared to WT, based on two biological replicates for each genotype. Histograms are divided into one plot for each chromosome. Chromosomal location is shown on the x-axis, scaled from 0 (centromere) to 1 (telomere); the y-axis shows Fold change of significantly changed peaks (FDR < 0.1). Graphs were produced using the R package ggplot2.
