# Supplementary material for: Endogenous mammalian histone H3.3 exhibits chromatin-related functions during development
Source: Epigenetics Chromatin. 2013 Apr 9;6:7. doi: 10.1186/1756-8935-6-7 (PMC3635903; doi:10.1186/1756-8935-6-7)
Supplement: Additional file 15 — Supplemental Methods. [file 1756-8935-6-7-S15.docx]

**Bush, et al. Supplemental Methods**

**Southern Blots**

For probes indicated below, the underlined regions were used as PCR primers for probe generation.

5’ probe *H3f3b* 519 bp

GGACCAACTGATGACCACCATACTTGAAAGGCTAACCTGAGTTACAGTCAAGTTTGGATGACGTCAGCCAACACTAGTCATTTGAAGAAACCAATACATTTTCTATAACCAATGTTCCCCTGTCCCAACTCCCAAAAGTGTTGAACCAGAGCTGCTTCTGCCTGACTTTAGTTCGGATGCTGCCAGCTCCTGGCTGGACGCTGGAGTGTTGTTCAATCTCTCGGGTTCTGACATTTCCACAGGTTGGGATCTCCTAAACTGGCCCATCACTCAGGCACCCAGTAGGGACACTCCTGGCACACAGCAGGGAACGGAAACCAACAGGGCTTTACAAGAGGAACTACAGCATTGGCCTTTGTCTCACAGAACCTAGGCCTACTGTGACATGACAGCAAGGAAGGCAGGGACCTAAGCTTGTACACATGCAGTTTTTACATTGTGGCAATCTCATTTAGGCCAGTTACGATGAGGCTCCCTTGCCTGCCCGATGCTGAAGGGGTCTGCAACCATTAACACCCC

3’ probe *H3f3b* 425bp

TGCAGTGGTGGTCAGCTCAGTGCCCCTGGGGGGTGGGCTTTCCAGCTCAGCGTCTCTGGAAGTGGCCACGTACACCTTCATCCAGCAGCTCTGCCCAGGTACCACTTAGCCGCCGCTTTCCCTCTCTGAGATCTCTGCGGCCAGAGCCTCTTCCTTTTCTTGGCTTAGGGAGGGAGGGTGGGGAGTCCCTCTGATTGTCAATGGAGCCCTGCTGCTCCAGGGTGGCCACGCCCCCCATGCCCTCTCCTGGACCCCCAGCTTCCCAACCTCACCCTGTGCTGCAGACTCGGGGGCAATAGCTGCCCGGGCCCAGGTGTGTCAACGGGCTGAGCACAGCTTCGCAGGGGTGCCCTGTGGCATCATGGACCAACTCATCGCGCTGCTGGGGCAGAAAGGCTATGCACTACTCATTGACTGCAGGTCAG

**ChIP-Seq**

ChIP-Seq was conducted with the following antibodies: to each diluted fraction was added either 2 ug anti- Acetylated Histone H3K9ac (Upstate 06-942), or 3 ul anti-H3K4me3 (Upstate 07-473) antiserum. After purification of the precipitated DNA and total input samples, gel size selection was conducted for 200-500 base fragments. Indexed ChIP-seq libraries were then prepared with the standard PrepX™ DNA Library Kit and the automated library preparation system Apollo324 (IntegenX). Four indexed libraries were pooled and sequenced in a single lane on the Illumina GAIIx platform producing short 40nt sequence reads. Fastq files were generated using the Illumina pipeline (CASSAVA 1.7). Indexed libraries were sorted into bins using the "barcoded data preparation tool" (http://comailab.genomecenter.ucdavis.edu/index.php/Barcoded_data_preparation_tools) and individual output files were aligned to the mm9 mouse genome (http://genome.ucsc.edu/cgi-bin/hgGateway?hgsid=220372099&clade=mammal&org=Mouse&db=0) using BOWTIE. SAM files were converted to bed6 files, which allows for peak calling using Sole-Search 2.0 ([[1](#_ENREF_1)]; http://chipseq.genomecenter.ucdavis.edu/cgi-bin/chipseq.cg)). Peaks were called using alpha-value 0.0001 and FDR 0.001 in combination with the histone and the blur length default of 1200 bp. The gff overlap tool of Sole-Search was used to compare peak lists. Peak calling was also performed using PeakRanger with the default settings, yielding very similar results [[2](#_ENREF_2)]. Identification of significantly changed ChIP-seq peaks was performed using the R package DiffBind, which uses the package edgeR for statistical analysis of differential binding.

Profiling of ChIP-Seq peaks around the TSS was performed using CEAS (<http://liulab.dfci.harvard.edu/CEAS/>) [[3](#_ENREF_3)]. Histograms of TSS-associated peaks and of chromosomal locations of peaks were generated in R using the ggplot2 package.

**Cellular Extracts**

Cellular Extracts were prepared from biological replicates of *H3f3b*^Δ/Δ^ and *H3f3b*^WT/WT^ MEFs. Cells were harvested, washed twice in 4°C PBS, and resuspended in 1X NuPage LDS Sample buffer. 1X NuPage buffer was prepared by diluting 4X LDS buffer (Cat no. NP0007) with dH_2_0. Samples were sonicated 1-1.5 minutes to shear DNA and frozen at -80°C.

**Embryo isolation/preparation**

*H3f3b*^Δ/WT^ mice were crossed, and embryos were isolated at E12.5 from 5 females. Embryos were rinsed thoroughly in PBS and fixed in 4% paraformaldehyde 4 hours – overnight, rinsed and stored in PBS. Embryos were then embedded in paraffin and sectioned at 7µm.

**Nuclear Size and Shape Analysis.**

Morphological parameters of DAPI-stained WT and KO nuclei were quantified from 20X photomicrographs using R with the image analysis package EBImage [[4](#_ENREF_4)]. At least 200 nuclei from each of two biological replicates were analyzed for each experimental group. Nuclear volume was estimated from surface area by assuming spherical nuclei. Shape factor (compactness) is defined as a ratio of the perimeter of the nucleus to the perimeter of a circle with the same area.

**Transduction-conditional KO**

At 20% confluency, two 10 cm plates of Plat-E packaging cells were transfected with 10µg of IRES-GFP or CRE-IRES-GFP vectors. Transfection reagents included 300µl of Opti-MEM (Gibco) and 30µl of Fugene HD (Roche) per vector. Medium was changed the following day, and we performed the transduction on the next day. The supernatant of virus producing Plat-E cells was carefully collected and filtered with 0.45µm filters. The viral solution was diluted 1:2 with fresh medium, polybrene was added at a final concentration of 5µg/ml, and the viral mix was added to 10 cm plates of *H3f3b*^Fl/Fl^ MEFs. Transduction was repeated the following day. Cells were harvested two days after the second transduction. Small portions of the cells were analyzed by flow cytometry to note GFP expression, which was greater than 95% in all samples (data not shown).

**RNA extraction**

RNA extraction protocols were identical for transduced *H3f3b*^Fl/Fl^ samples and for constitutive *H3f3b*^WT/WT^, *H3f3b*^Δ/WT^ and *H3f3b*^Δ/Δ^ E12.5 MEFs. 10 cm plates were harvested, washed once with 1x PBS and pellets were processed using High Pure RNA Isolation kit (Roche), and corresponding protocol. RNA was then nanodropped and 1-3µl was used for cDNA production. cDNA was prepared using iScript cDNA Synthesis kit (Bio-Rad) and corresponding protocol.

**qPCR analysis**

Primers used for Sybr green analysis are as follows:

CENPA - CGAGGCTTCGAGGGCGGACT; TCTGTGTCGGGGGAAGGCCA

CDC20 - CTGTGGCATGGTGTCCCTGGC; GCTGGTTCTGGGCAAAGCCGT

CCND2 - AGTGGAACCTGGCCGCAGTC; CGACGGCGGGTACATGGCAAA

BIRC5 - CAGTGAGGAAGGCGCAGCCAG; ACCCCAGAGCGAATGGCGGA

DBF4 - AATATGGACGGCGATAAATGTGG; CTGTGTTTCTCACTCAGAAGGTG

MCM5 - TGAACTCAAGCGGCATTACAA; GGCTGTTTATGCAAGTGGTCA

RRM1 - AGGTTGCAGTTTGTAACTTGGC; GGCCGATGGCGTTTATTTGAT

ORC6I - CCTCTGGTCGCAACCAATCT; TTCACCTTAGACAAGCCGCAG

**Comparison of histone peak changes in H3.3 KOs with CTCF peaks from existing data sets.**

GEO dataset GSM918743, which is Chipseq data for the insulator CTCF in MEFs, was used. We compared the CTCF peak locations to the H3K4me3 and H3K9ac peaks in WT and KO MEFs. The CTCF data is available as pre-called peaks (aligned to the mm9 genome) in broadPeak format (BED6+3), which was converted to GFF format in Galaxy so that it matched the format of our MEF data. We examined two sets of peaks from our data: the peaks that overlap between WT and KO (shared peaks), and peaks that don't overlap between WT and KO (unique peaks). We did the analysis using the GFF-Overlap tool from Sole Search in two different ways: first by looking for peaks within 1000bp of each other, then by looking for peaks that are directly touching. We obtained essentially the same results both ways. To determine the statistical significance, we made 2x2 contingency tables for the overlap of each set of peaks with the CTCF peaks, then we did a Chi-squared test for each contingency table.

**Image analysis and Z-stack capture.**

MEFs treated with antibodies were imaged on a Nikon C1 confocal microscope with a motorized stage utilizing Nikon TE/EZ capture and controller software. All Z-stack images were captured using a Plan Apo 60X oil immersion objective lens. MEF nuclei were captured at random under DAPI, and an average of 17 Z-stack images beginning from the bottom and continuing to the top of the nuclei were taken at 0.5um steps (approximately 8-9um total). Acquisition of images was done using identical camera gain, exposure time, and laser settings between *H3f3b* KO and *H3f3b* WT MEF captures. Images were saved in .avi format and analyzed using ImageJ software (Rasband W.S., U. S. National Institutes of Health, Bethesda MD) [[5](#_ENREF_5)]. Two *H3f3b* KO and two *H3f3b* WT MEF lines were analyzed in two separate experiments (a total of n = 4 *H3f3b* KO and *H3f3b* WT analyses). Approximately 80-100 nuclei were analyzed per experiment using identical ImageJ program settings. ImageJ was used to calculate nuclear area, circularity, and regions of interest (ROI) for the measurement of kinetochore foci and mean fluorescence intensities (MFIs).

**ImageJ Cell Foci, MFI, and Cellular Analysis**

ImageJ software (Version 1.46R, Rasband WS, National Institutes of Health, Bethesda MD; Schneider CA *Nature* 2012) was used utilizing built-in plugins in addition to the Cell Counter plugin (Kurt De Vos, University of Sheffield, Academic Neurology, UK) for manual counting of cells and kinetochore foci. Tri-color Z-stack images (DAPI/DNA, blue; FITC/CREST serum, green; TRITC/CENP-A, red) .avi images were loaded into ImageJ and separated into 8-bit color channels using the *Split Channels* function (found in the *Color* tab under the *Image* menu). DAPI grayscale Z-stacks were then used to generate a grayscale projection containing the sum of all DAPI images within the Z-stack of images in order to calculate the maximum possible area in which kinetochore foci or kinetochore staining could be found within nuclei (utilizing the *Z Project* function in the *Stacks* tab under the *Image* menu [[6-8](#_ENREF_6)]). DAPI Z-stack projections were then used to calculate nuclear area, circularity, and regions of interest (ROI) using the *Analyze Particles* function (found under the *Analyze* menu). This method was checked manually and produced accurate measurements. Thresholding using the *Analyze Particles* function excluded small, fragmented nuclei and selected for nuclei with 0.58-1.00 circularity, where 0.00 represents a straight line, 0.50 represents an elongated nuclei, and 1.00 represents a perfectly circular nuclei. To identify foci within the nuclear ROIs, CREST/CENP-A Z-stack grayscale images were then analyzed using the *Find Maxima* function (found under the *Process* menu) in order to account for possible photobleaching in the Z-stacks. This function returned data points from each individual Z-stack image on the number of local maximum intensities that stood above background noise for the measurement of individual foci. The *Find Maxima* method of quantitating foci was checked manually using the *Cell Counter* plugin to count foci on each individual Z-stack section, and was found to produce accurate measurements. Noise tolerance within this function was adjusted to produce accurate measurements of foci over background noise; identical noise tolerance settings were used between *H3f3b* KO and WT nuclei. To identify the total CREST/CENP-A signal within nuclei, CREST/CENP-A Z-stack grayscale images were used to generate a grayscale projection (utilizing the *Z Project* function) containing the sum of all the CREST or CENP-A images within a Z-stack in order to calculate the total CREST/CENP-A MFI within a nuclei. ROIs from the DAPI Z-stack projections were used to obtain MFI measurements in *H3f3b* KO and WT nuclei.

**Supplemental References**

1. Blahnik KR, Dou L, Echipare L, Iyengar S, O'Geen H, Sanchez E, Zhao Y, Marra MA, Hirst M, Costello JF, et al: **Characterization of the contradictory chromatin signatures at the 3' exons of zinc finger genes.** *PLoS One* 2011, **6:**e17121.

2. Feng X, Grossman R, Stein L: **PeakRanger: a cloud-enabled peak caller for ChIP-seq data.** *BMC bioinformatics* 2011, **12:**139.

3. Shin H, Liu T, Manrai AK, Liu XS: **CEAS: cis-regulatory element annotation system.** *Bioinformatics* 2009, **25:**2605-2606.

4. Pau G, Fuchs F, Sklyar O, Boutros M, Huber W: **EBImage--an R package for image processing with applications to cellular phenotypes.** *Bioinformatics* 2010, **26:**979-981.

5. Schneider CA, Rasband WS, Eliceiri KW: **NIH Image to ImageJ: 25 years of image analysis.** *Nature methods* 2012, **9:**671-675.

6. Santenard A, Ziegler-Birling C, Koch M, Tora L, Bannister AJ, Torres-Padilla ME: **Heterochromatin formation in the mouse embryo requires critical residues of the histone variant H3.3.** *Nature cell biology* 2010, **12:**853-862.

7. Mukhopadhyay A, Elattar A, Cerbinskaite A, Wilkinson SJ, Drew Y, Kyle S, Los G, Hostomsky Z, Edmondson RJ, Curtin NJ: **Development of a functional assay for homologous recombination status in primary cultures of epithelial ovarian tumor and correlation with sensitivity to poly(ADP-ribose) polymerase inhibitors.** *Clinical cancer research : an official journal of the American Association for Cancer Research* 2010, **16:**2344-2351.

8. Cai Z, Vallis KA, Reilly RM: **Computational analysis of the number, area and density of gamma-H2AX foci in breast cancer cells exposed to (111)In-DTPA-hEGF or gamma-rays using Image-J software.** *International journal of radiation biology* 2009, **85:**262-271.
